# Proteogenomic characterization of cervical cancer identifies molecular subtypes predictive of clinical outcomes and subtype-specific targets

**DOI:** 10.1172/JCI199497

**Published:** 2026-02-10

**Authors:** Xun Tian, Mansheng Li, Zhi Wang, Tian Fang, Yi Liu, Jin Fang, Lejing Wang, Zhichao Jiang, Xingyu Zhao, Chen Cao, Zhiqiang Yu, Meiying Yang, Songfeng Wu, Yifan Wu, Rui Tian, Hui Wang, Yunping Zhu, Zheng Hu

**Affiliations:** 1 Department of Obstetrics and Gynecology, Academician Expert Workstation, The Central Hospital of Wuhan, Tongji Medical College, Huazhong University of Science and Technology, Wuhan, Hubei, China.; 2 State Key Laboratory of Medical Proteomics, Beijing Proteome Research Center, National Center for Protein Sciences (Beijing), Beijing Institute of Lifeomics, Beijing, China.; 3 Department of Gynecology, Guilin People’s Hospital, Guilin, Guangxi, China.; 4 Beijing Qinglian Biotech Co. Ltd, Beijing, China.; 5 Generulor Company Bio-X Lab, Zhuhai, Guangdong, China.; 6 Department of Gynecologic Oncology, Women’s Hospital, Zhejiang University School of Medicine, Hangzhou, Zhejiang, China.; 7 Zhejiang Provincial Key Laboratory of Precision Diagnosis and Therapy for Major Gynecological Diseases, Women’s Hospital, Hangzhou, Zhejiang, China.; 8 Taikang Center for Life and Medical Sciences, Wuhan University, Wuhan, Hubei, China.; 9 Department of Gynecologic Oncology, Women and Children’s Hospital Affiliated to Zhongnan Hospital of Wuhan University, Wuhan, Hubei, China.; 10 School of nursing, Research Center for Lifespan Health, Wuhan University, Wuhan, Hubei, China.; 11 Hubei Key Laboratory of Tumor Biological Behavior, Hubei Provincial Clinical Research Center for Cancer, Zhongnan Hospital of Wuhan University, Wuhan, Hubei, China.

**Keywords:** Genetics, Oncology, Virology, Cervical cancer, Molecular diagnosis, Proteomics

## Abstract

**BACKGROUND:**

Cervical cancer (CC) remains the fourth leading cause of cancer-related deaths in women globally, with poor prognosis for metastatic and recurrent cases. Although genomic alterations have been extensively characterized, global proteogenomic landscape of the disease is largely under explored.

**METHODS:**

Here, we present the first genome-wide proteogenomic characterization of CC, analyzing 139 tumor-normal tissue pairs using whole-genome sequencing, transcriptomics, proteomics, and phosphoproteomics.

**RESULTS:**

We identified 4 distinct molecular subtypes with unique clinical outcomes: epithelial-mesenchymal transition (EMT, C1), proliferation (C2), immune response (C3), and epithelial differentiation (C4). A 4-protein classifier (CDH13, TP53BP1, NNMT, HSPB1) was developed with strong prognostic and predictive value, particularly for immunotherapy response in subtype C3. Phosphoproteomic profiling uncovered subtype-specific kinase activity, identifying actionable therapeutic targets.

**CONCLUSION:**

Our findings further revealed previously uncharacterized somatic copy number alterations, extrachromosomal DNA landscape, and human-HPV fusion peptides, with implications for genetic heterogeneity and therapeutic targets. This study enhances the understanding of cervical cancer through deeper proteogenomic insights and facilitates the development of personalized therapeutic strategies to improve patient outcomes.

**FUNDING:**

Noncommunicable Chronic Diseases-National Science and Technology Major Project (2025ZD0544102);The National Natural Science Foundation of China (82172584); Key Technology R&D Program of Hubei (2024BCB057 and 2025BCB053); National Natural Science Foundation of China (82373260); the “4+X” clinical trial programs of Women’s Hospital, School of Medicine, Zhejiang University (LY2022004); and the programs of Zhejiang Traditional Chinese Medicine Innovation Team (CZ2024009); and Guangxi Natural Science Foundation (2024GXNSFBA010045).

## Abstract 2

Researchers identified four cervical cancer subtypes by mapping proteins and genes, enabling personalized treatments and better predictions of patient outcomes and therapy responses.

## Introduction

Cervical cancer (CC) stands as the most predominant cancer type associated with human papillomavirus (HPV) (1). Despite screening strategies reducing the incidence and mortality of CC in high-income countries, the global burden remains substantial with over 300,000 deaths per year worldwide (2). Particularly in Asia, where vaccination and screening coverages are low due to large populations, nearly 200,000 deaths occur annually (2). Current treatments for CC have not substantially improved the prognosis, especially for metastatic and recurrent cases with 5-year survival limited to 15%–20% (3, 4). This highlights the critical urgency for a comprehensive understanding of the molecular mechanisms underlying the initiation and progression of CC (5–7). Acquiring such knowledge is essential for identifying targets that could potentially transform therapeutic strategies and improve patient outcomes (8–10).

In the past decade, genomic studies have markedly advanced our understanding of the molecular underpinnings of CC (11–13). Comprehensive proteogenomic studies have begun to bridge the gap between genomic alterations and their downstream protein-level consequences, revealing subtype-specific signaling pathways, therapeutic vulnerabilities, and mechanisms of treatment resistance (14, 15). Nevertheless, these pioneering efforts were still limited by cohort composition, cancer stage representation, or the depth of proteomic characterization, leaving important aspects of how multilayered molecular changes shape phenotypic diversity and clinical outcomes in insufficiently resolved CC (16, 17). Therefore, systematic multi-omics investigations remain essential to delineate how genomic alterations remodel the proteome and functional networks that drive cervical cancer progression and therapeutic response.

This study presents the first genome-wide proteogenomic characterization of CC, analyzing a cohort of 139 treatment-naive CC specimens and their paired normal adjacent tissues (NATs). Through an integrated analysis of whole-genomic, transcriptomic, proteomic, and phosphoproteomic data, our research aims to elucidate the molecular landscape of CC, refine molecular subtyping, identify prognostic biomarkers, and uncover potential therapeutic targets.

## Results

### Genomic, proteomic, and phosphoproteomic landscapes of CC.

**Figure 1:**
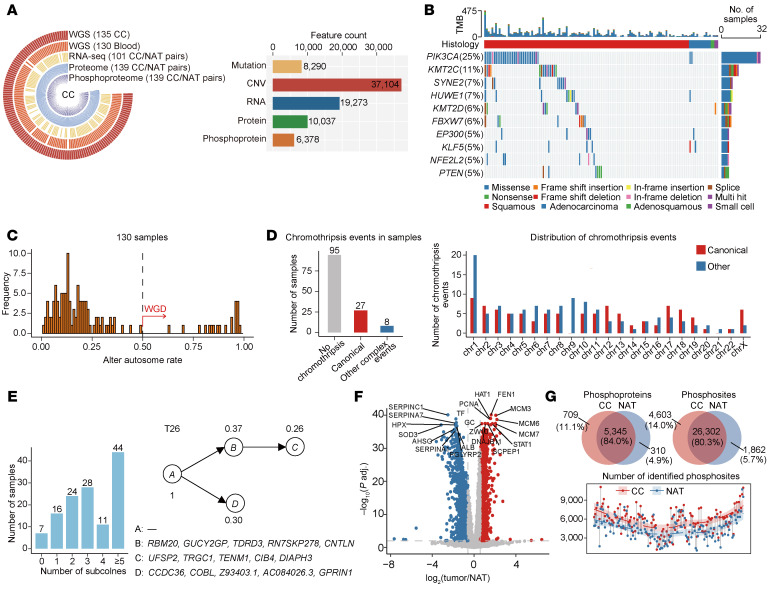
Multi-omics landscape of CC. (**A**) Overview of data types and quantified features. (**B**) Genetic profile of the 129 patients with CC, ordered by histology and mutation frequency. (**C**) Bimodal distribution of the fraction of the autosomal genome. Samples were considered to have undergone WGD if more than 50% of their autosomal genome had major integer copy number > 2. (**D**) Bar plot showing the number of samples harboring chromothripsis events (left) and chromothripsis events across chromosomes (right). (**E**) Number of subclonal populations per tumor (left). Representative subclonal structure of tumor T26 reconstructed by PhyloWGS (right). (**F**) Volcano plot illustrating differentially expressed proteins between tumors and NATs (Benjamini-Hochberg adjusted *P* < 0.01, fold change > 1.5, modified *t* test), upregulated (red) and downregulated (blue) proteins are shown. Top 10 most significantly altered proteins with the lowest adjusted *P* value are labeled. (**G**) Venn diagram illustrating overlap of identified phosphoproteins (top-left) and phosphosites (top-right) in tumors and NATs; bottom panel indicates the number of phosphosites identified in tumors and matched NATs annotated by grey straight lines.

To delineate the genomic landscape of CC, we conducted whole-genome sequencing (WGS) on 130 CC tissue samples and matched blood specimens (Figure 1A, Supplemental Note 1, Supplemental Figure 1, and Supplemental Table 1; supplemental material available online with this article; https://doi.org/10.1172/JCI199497DS1). Recurrently mutated genes included *PIK3CA* (25%), *KMT2C* (11%), *SYNE2* (7%), *HUWE1* (7%), *KMT2D* (6%), *FBXW7* (6%), *EP300* (5%), *KLF5* (5%), *NFE2L2* (5%), and *PTEN* (5%) (Figure 1B and Supplemental Table 2).

Next, we identified whole-genome doubling (WGD) events in 16% (21 of 130) of cases (Figure 1C), and detected high-confidence chromothripsis (defined by the presence of greater than or equal to 7 oscillating segments) in 27% (35 of 130) of cases. Among these, 27 samples showed canonical chromothripsis events (Figure 1D left), predominantly affecting chromosomes 1, 2, 12, and 17 (Figure 1D right). Moreover, phylogenetic reconstruction revealed extensive intratumor heterogeneity, with 82% (107 of 130) of cases harboring greater than or equal to 2 clonally distinct subpopulations (Figure 1E). Collectively, these findings highlighted the frequent occurrence of WGD, chromothripsis, and substantial intratumor heterogeneity in CC, all of which contribute to increased genomic complexity and branched evolutionary trajectories.

We next conducted global proteomic and phosphoproteomic in 139 paired CC and normal adjacent tissues (NATs), identifying a total of 10,037 proteins (Supplemental Figure 2). Among these, the downregulated proteins (*n* = 1,041) were predominantly associated with complement activation and humoral immune response, revealing a suppressed immune environment during cervical carcinogenesis (Figure 1F and Supplemental Figure 3A). In contrast, the upregulated proteins (*n* = 1,706) were enriched in RNA processing pathways, including mRNA metabolism, ribonucleoprotein complex biogenesis, translation, and RNA splicing (Figure 1F and Supplemental Figure 3B). Of the upregulated proteins, we further stratified 23 proteins with more than a 1.5 Log_2_ fold change in at least 90% of tumors (Supplemental Figure 3C). Among them, TACSTD2, TFRC, and TYMP are targeted by Food and Drug Administration (FDA) approved drugs, and have potential for immediate clinical translation (Supplemental Note 2).

The phosphoproteome profiling of 139 tumor/NAT pairs revealed a total of 32,767 phosphosites corresponding to 6,364 phosphoproteins with a confident site location score (probability greater than 0.75). Among these phosphosites, 11,443 were previously documented in established databases (18), including 1,731 sites with kinase information, 1,529 with regulatory information, and 1,241 possessing both kinase and regulatory details (Figure 1G). The number of phosphosites identified in the tumors strikingly exceeded that in the paired NATs (*P* = 4.53×10^–14^, Wilcoxon signed-rank test; Figure 1G).

### Integrated proteogenomic analysis of somatic copy number alterations reveals previously uncharacterized drivers and prognostic biomarkers.

**Figure 2:**
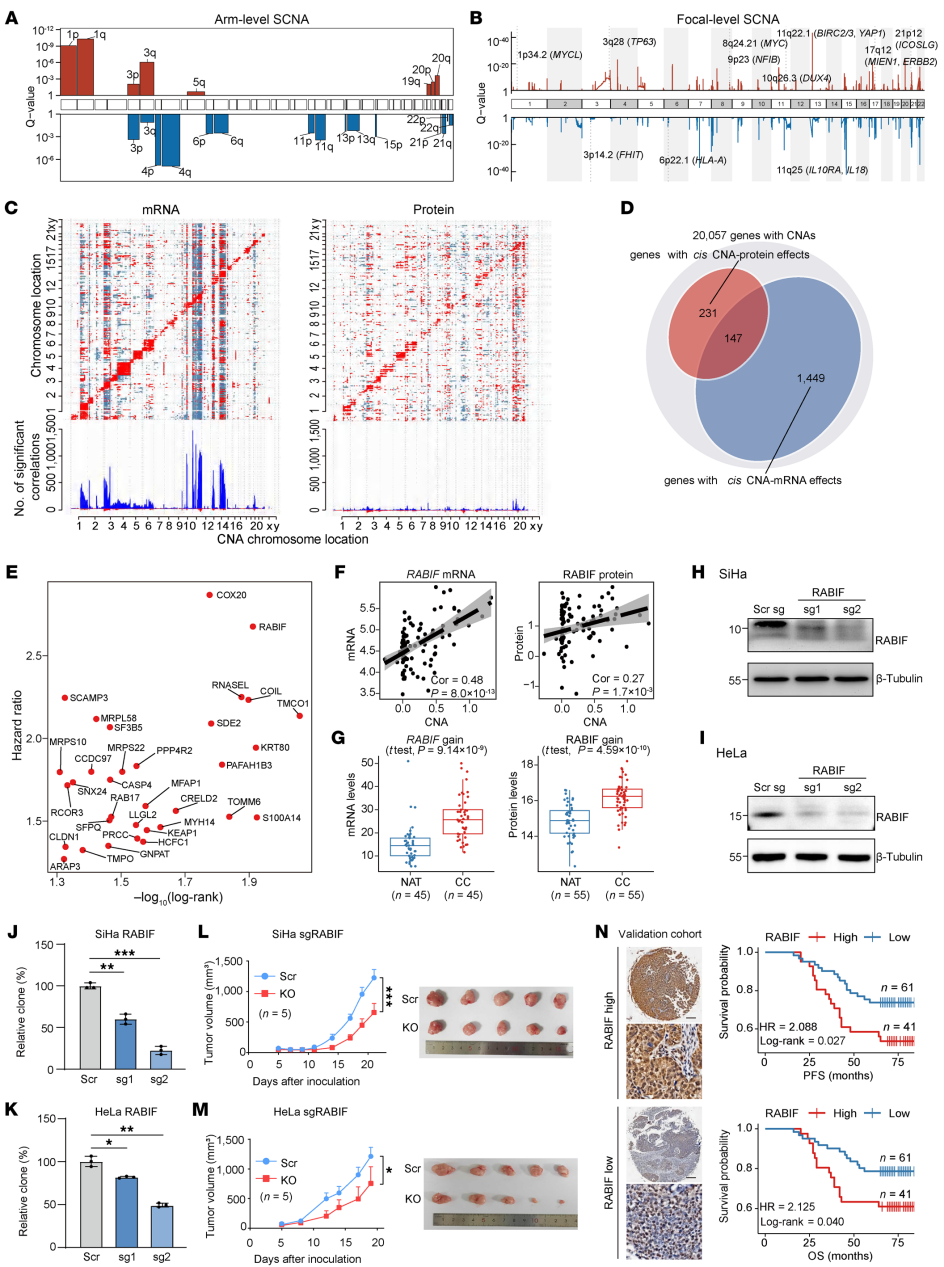
The impacts of SCNAs on mRNA and proteins. (**A** and **B**) Significant arm-level and focal-level SCNA events (Q-value < 0.25) in patients with CC. (**C**) Heatmaps show significant positive (red) and negative (blue) Spearman’s correlations (Benjamini-Hochberg *P_adj_* < 0.001) between CNAs and mRNA (left) or protein (right) levels. (**D**) Number of CNAs with significant cis effects (*P* < 0.01, Spearman’s correlation) on mRNA alone, or protein alone, or both. (**E**) Scatterplot showing proteins significantly associated with patient overall survival (OS) (*P* < 0.05, Log-rank test) and upregulated in tumors (Benjamini-Hochberg *P_adj_* < 0.01, fold change > 1.5, modified *t* test). (**F**) Spearman’s correlations between *RABIF* CNA and mRNA (left) or protein (right) abundances. (**G**) Box plot showing *RABIF* mRNA and protein levels in copy number gain CC samples versus paired NATs (2-sided Student’s *t* test). Centers indicate the medians, the upper and lower boundaries of the boxes indicate the 75th and 25th percentile, whiskers extend to 1.5× IQR. (**H** and **I**) Western blot analysis of *RABIF* knockout efficiency in SiHa (**H**) and HeLa (**I**) cells (2 biological replicates). (**J** and **K**) Colony formation assays in SiHa (**J**) and HeLa (**K**) cells following *RABIF* knockout. Data represent means ± SEM (*n* = 3 biological replicates, 1-way ANOVA with Tukey’s multiple comparisons test). (**L** and **M**) Tumor growth curves in xenograft models of SiHa (**L**) and HeLa (**M**) cells with *RABIF* knockout. Data represent mean ± SEM (*n* = 5 mice per group, two-way analysis of variance). (**N**) Representative IHC images of RABIF-high (top-left) and RABIF-low (bottom-left) expression in an external patient cohort (*n* = 102). Scale bars: 200 μm. Kaplan-Meier curves for PFS and OS are shown. Statistical significances were determined by Log-rank test. *P < 0.05, **P < 0.01, ***P < 0.001.

To comprehensively characterize somatic copy number alterations (SCNAs) in CC, we analyzed chromosomal arm-level events using GISTIC2.0 (19), which revealed frequent gains on 1p, 1q, 3p, 3q, 5q, 19q, 20p, and 20q, alongside losses on 3p, 3q, 4p, 4q, 6p, 6q, 11p, 11q, 13p, 13q, 15p, 21p, 21q, 22p, and 22q (Figure 2A and Supplemental Figure 4A). Among these, chromosome 1q gain emerged as the most significant (Q-value = 5.29 × 10^–11^) and frequently observed event (28%) across the 130 CC samples. Further, we identified 112 focal gains and 90 focal losses (Q-value < 0.25; Supplemental Table 2). Consistent with previously reports (5, 20–22), we confirmed recurrent gains at 3q28 (*TP63*; 72%), 8q24.21 (*MYC*; 35%), 11q22.1 (*BIRC2*, *BIRC3*, *YAP1*; 11%), and 17q12 (*ERBB2*, *MIEN1*; 8%), as well as recurrent losses at 3p14.2 (*FHIT*; 45%) and 11q25 (*IL18*, *IL10RA*; 62%) (Figure 2B and Supplemental Figure 4B). Beyond these well-characterized regions, we also discovered previously uncharacterized recurrent gains at 1p34.2 (*MYCL*; 13%), 9p23 (*NFIB*; 17%), 10q26.3 (*DUX4*; 6%), and 21p12 (*ICOSLG*; 53%), and a recurrent loss at 6p22.1 (*HLA-A*; 25%). These findings were further validated through the FACETS algorithm (Supplemental Figure 4C).

To explore the downstream molecular events of SCNAs, we examined cis-regulatory effects by correlating copy number changes with gene expression levels (Figure 2C). This analysis identified 1,449 significant CNA-mRNA correlations and 231 CNA-protein correlations (Spearman’s correlation test, *P* < 0.001; Figure 2D and Supplemental Table 3). A subset of 147 genes exhibited consistent cis-effects at both the mRNA and protein levels (Figure 2D). Among them, *RABIF* and *SCAMP3* were markedly upregulated in tumors and associated with poor prognosis (Figure 2E). In tumor samples carrying *RABIF* gain, both mRNA and protein expression were concordantly elevated (Figure 2, F and G). To validate *RABIF* as potential driver CNAs and prognostic indicator, we performed functional assays and prognostic analysis using a CC tissue microarray (TMA) cohort (*n* = 102). Experimental assays showed that knockout of *RABIF* in SiHa and HeLa cells markedly suppressed colony formation (Figure 2, H–K) and attenuated tumor growth in xenograft models (Figure 2, L and M). Mechanistic investigation showed that *RABIF* knockout led to reduced RAB10 protein levels (Supplemental Figure 5, A and B) and impaired cellular glucose uptake (Supplemental Figure 5C). Given the established role of RAB10 in glucose transport (20), these results indicated that *RABIF* maintains glucose metabolism by regulating RAB10 protein levels. Clinically, elevated RABIF expression was correlated with shorter progression-free survival (PFS) (HR = 2.088, *P* = 0.027, Log-rank test) and overall survival (OS) (HR = 2.125, *P* = 0.04, Log-rank test; Figure 2N and Supplemental Table 3) in the independent validation cohort. Similarly, gain of *SCAMP3* enhanced EGFR–AKT signaling to promote tumor growth and elevated SCAMP3 expression was associated with poor patient survival (Supplemental Note 3, Supplemental Figure 6, and Supplemental Table 3).

### Proteogenomic clustering defines 4 molecular subtypes with distinct clinical outcomes and biological characteristics.

**Figure 3:**
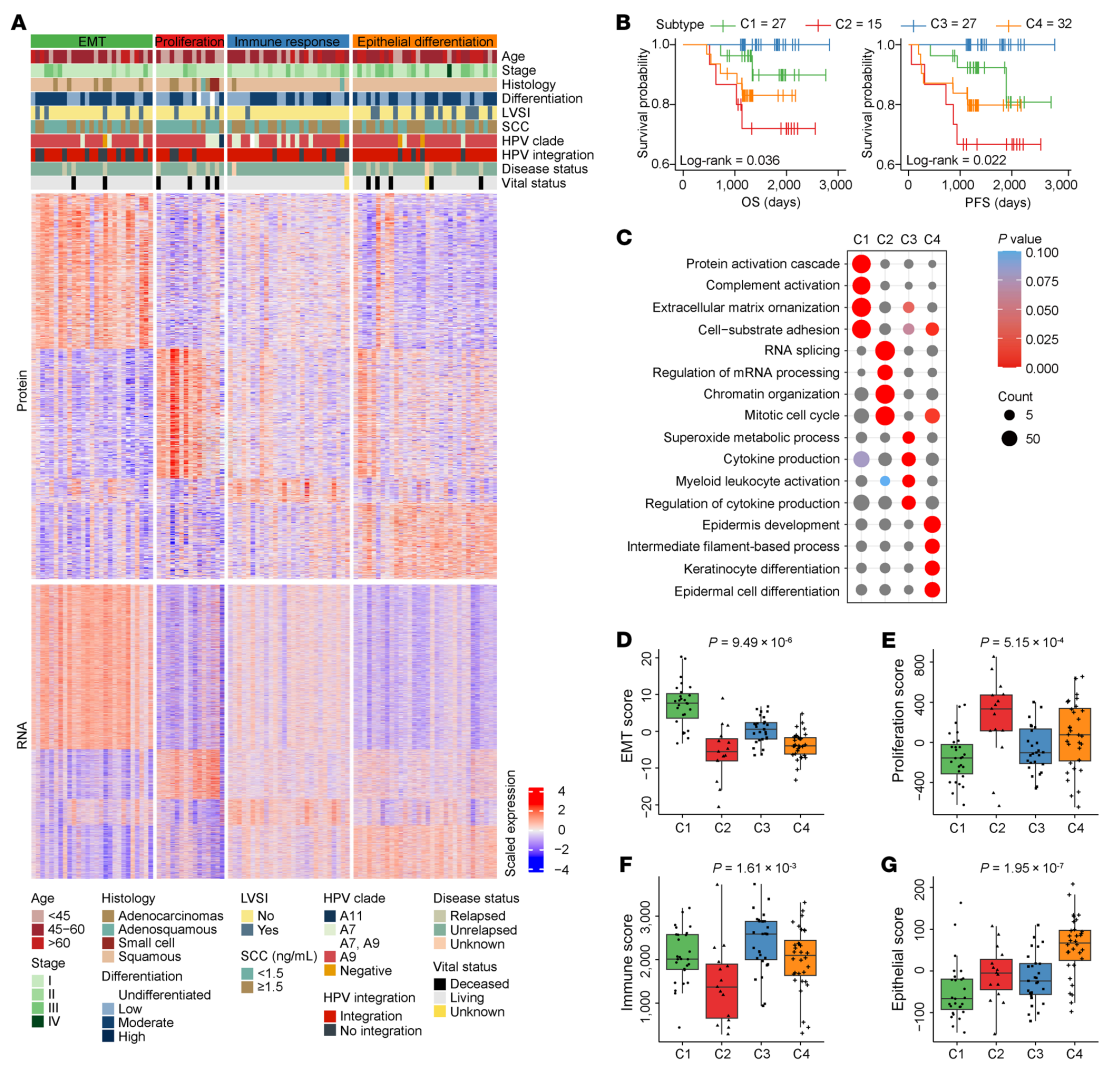
Multi-omics clustering identified 4 CC molecular subtypes with prognostic relevance. (**A**) SNF-CC analysis of 101 patients with CC using transcriptomic and proteomic data identified 4 distinct molecular subtypes. Clinicopathological features are annotated. (**B**) Kaplan-Meier curves comparing OS (left) and PFS (right) across all subtypes (Log-rank test). (**C**) GO-BP terms enriched for subtype-special proteins. (**D**–**G**) Subtype-wise comparisons of EMT score (**D**), proliferation score (**E**), immune score (**F**), and epithelial score (**G**), analyzed using 1-way ANOVA. In each box plot, the center line shows the median, the box limits indicate the upper and lower quartiles, and the whiskers extend to 1.5× IQR.

Using ensemble Similarity Network Fusion (SNF) and Consensus Clustering algorithm to proteogenomic profiles from 101 CC samples, we identified 4 distinct molecular subtypes (C1–C4) (Figure 3A). These subtypes exhibited distinct clinical outcomes, with significant differences in OS (*P* = 0.036, Log-rank test) and PFS (*P* = 0.022, Log-rank test), where C3 showed the most favorable prognosis and C2 the poorest (Figure 3B).

Proteogenomic pathway analysis revealed subtype-specific biological characteristics (Figure 3C and Supplemental Table 4). C1 showed enrichment in extracellular matrix organization and wound healing pathways, with elevated EMT score (Figure 3D). C2 exhibited upregulation of RNA processing pathways and elevated proliferation scores (Figure 3E). C3 demonstrated robust immune score (Figure 3F) and was enriched in immune response signatures including leukocyte activation and cytokine production pathways. C4 was distinguished by its high epithelial differentiation score (Figure 3G) and showed enrichment in epithelial differentiation pathways, with a particularly higher frequency of lymphovascular space invasion (LVSI) (Supplemental Figure 7). Accordingly, we designated the subtypes as the EMT (C1), proliferation (C2), immune response (C3), and epithelial differentiation (C4). The expression patterns of subtype-specific genes were presented in Supplemental Note 4 and Supplemental Figure 8. Our molecular classification system was validated through TCGA cohort analysis, demonstrating concordance with established subtypes while revealing clinically relevant subgroups, particularly the high-risk proliferative C2 and immune-active C3 subtypes, which were previously uncharacterized (Supplemental Note 5 and Supplemental Figure 9).

**Figure 4:**
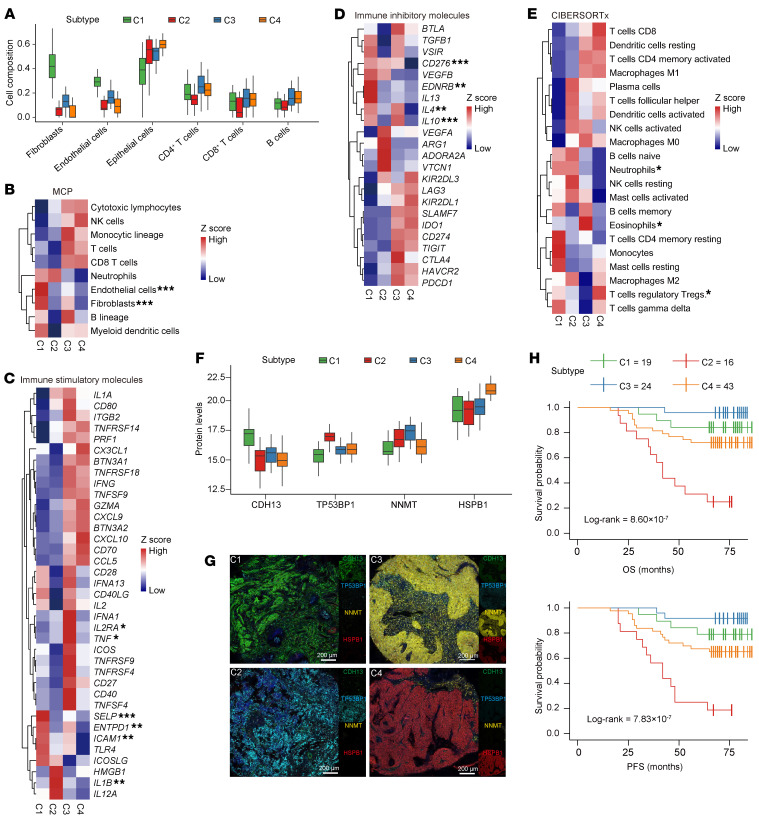
Characteristics of CC molecular subtypes and validation in independent cohorts. (**A**) Cell type compositions derived from xCell analysis of transcriptomic data. Centers indicate the medians, the upper and lower boundaries of the boxes indicate the 75th and 25th percentile, whiskers extend to 1.5× IQR. (**B**) Heatmaps showing the average immune cell populations in each subtype, inferred from transcriptomic data using MCPcounter. (**C** and **D**) Heatmaps showing the average mRNA expression of immune stimulatory molecules (**C**) and immune inhibitory molecules (**D**) across subtype. (**B**) Heatmaps showing the average immune cell populations in each subtype, inferred from transcriptomic data using CIBERSORTx. Differences between the C3 and C4 subtypes were assessed using the Wilcoxon rank-sum test adjusted by Benjamini-Hochberg in **B**–**E**. *P_adj._ < 0.05, **P_adj._ < 0.01, ***P_adj._ < 0.001. (**F**) Box plot showing protein expression levels of subtype-specific markers across the 4 subtypes. Centers indicate the medians, the upper and lower boundaries of the boxes indicate the 75th and 25th percentile, whiskers extend to 1.5× IQR. (**G**) Representative mIF staining images illustrating the molecular features of 4 proteomic subgroups. Scale bars: 200 μm. (**H**) Kaplan-Meier curves for OS (left) and PFS (right) based on the mIF staining of 4 subgroup-specific biomarkers in an external independent CC cohort (*n* = 102). Log-rank tests are used for statistical analysis.

To investigate tumor cell composition and its relationship with molecular subtypes, we performed cellular deconvolution and immune profiling (Method; Immune score and immune cell type composition). The EMT subtype (C1) and proliferation subtype (C2) exhibited noninflamed, immune-cold characteristics with minimal T cell infiltration. C1 was characterized by low immune infiltration and the highest abundance of fibroblasts and endothelial cells, while C2 tumors displayed low presence of both immune and stromal cells (Figure 4, A and B). In contrast, the immune response subtype (C3) represented an immune-hot phenotype, featuring robust CD8^+^ T cell infiltration (Figure 4A and Supplemental Table 4) and elevated expression of immune-stimulatory molecules and immune checkpoint targets (Figure 4, C and D). The epithelial differentiation subtype (C4) was enriched in epithelial cells (Figure 4A) and was characterized by a higher prevalence of regulatory T cells (Tregs) (Figure 4E). The immune and stromal cell abundances were further validated by immunohistochemistry (IHC) for key markers (α-SMA, CD4, CD8, and CD68) (Cor > 0.55, *P* < 0.05; Supplemental Figure 10).

We used the TMA cohort (*n* = 102) to evaluate the robustness of our proteogenomic subtyping. Using least absolute shrinkage and selection operator (LASSO) regression, we developed a 4-protein classifier comprising CDH13 (C1), TP53BP1 (C2), NNMT (C3), and HSPB1 (C4) (Figure 4F and Supplemental Table 4). Using multiplex immunofluorescence (mIF), this 4-protein classifier effectively stratified the independent TMA cohort into subtypes C1–C4 (Figure 4G). These classifier-defined subtypes displayed distinct OS (*P* = 8.6 × 10^–7^, Log-rank test) and PFS (*P* = 7.83 × 10^–7^, Log-rank test) (Figure 4H and Supplemental Table 4), successfully recapitulating the prognostic trends observed in our proteogenomic molecular subtyping.

### Validation of subtype-specific targets and a 4-protein classifier for precision therapy in CC.

**Figure 5:**
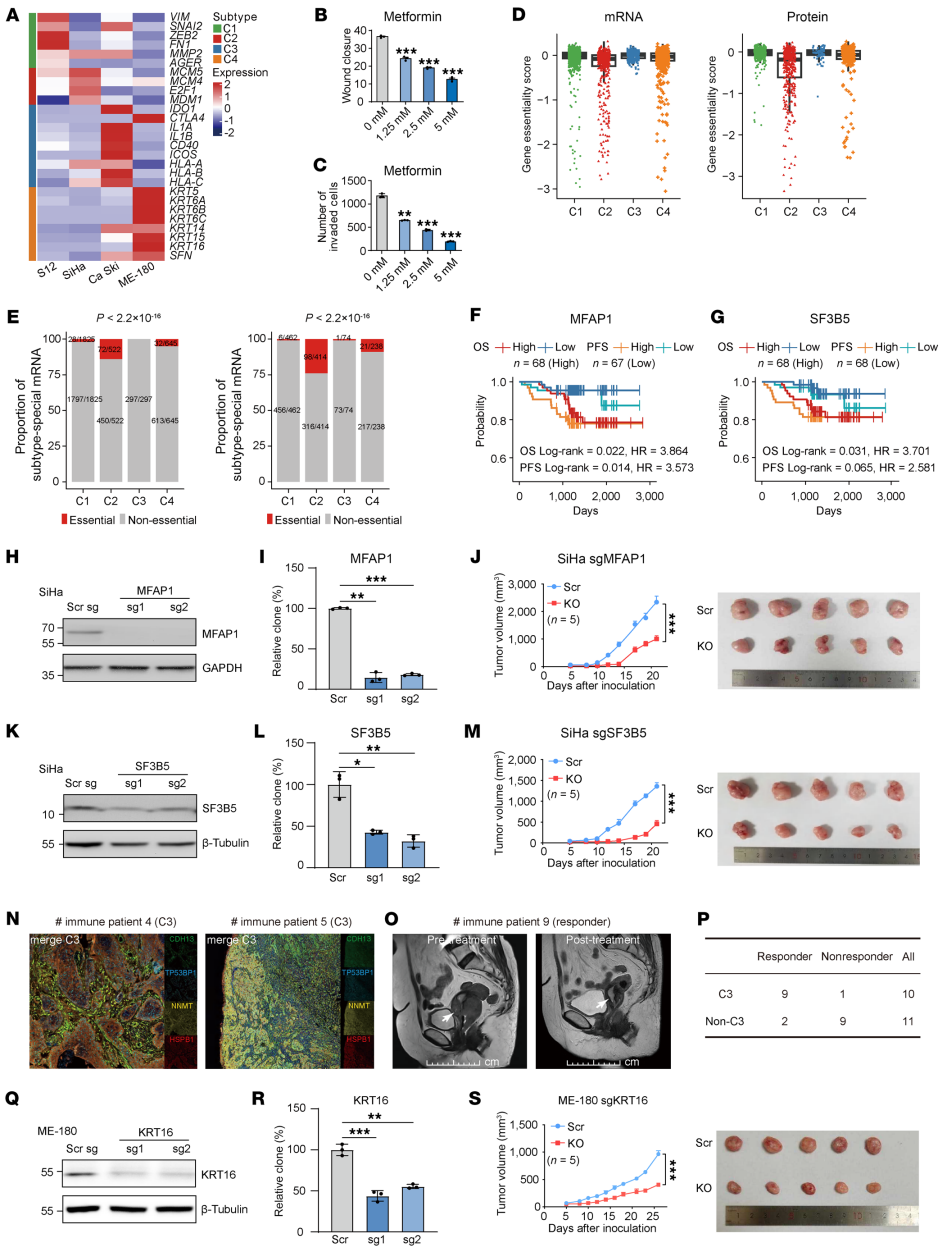
Validation of CC molecular subtypes through experimental assays. (**A**) mRNA expression of subtype-associated genes across 4 CC cell lines. (**B** and **C**) Quantification of transwell migration (**B**) and wound healing (**C**) in S12 cells (*n* = 3 biological replicates) treated with metformin. Data represent means ± SEM (2-sided Student’s *t* test). (**D**) Gene essentiality scores of subtype-special mRNAs (left) and proteins (right). Centers indicate the medians, the upper and lower boundaries of the boxes indicate the 75th and 25th percentile, whiskers extend to 1.5× IQR. (**E**) Distribution of essential genes (gene essentiality score < –0.7) across subtypes (χ^2^ test). (**F** and **G**) Kaplan-Meier curves for OS and PFS of patients stratified by median protein abundance of MFAP1 (**F**) and SF3B5 (**G**) (Log-rank test). (**H**, **K**, and **Q**) Validation of knockout efficiency for each sgRNA targeting *MFAP1* (**H**), *SF3B5* (**K**), and KRT16 (**Q**) by Western blot analysis (*n* = 2 biological replicates). (**I**, **L** and **R**) The impacts of *MFAP1* (**I**), *SF3B5* (**L**), and *KRT16* (**R**) knockout on colony formation in SiHa cells. Data represent means ± SEM (*n* = 3 biological replicates, 1-way ANOVA with Tukey’s multiple comparisons test). (**J**, **M**, and **S**) The impacts of *MFAP1* (**J**), *SF3B5* (**M**), and KRT16 (**S**) knockout on xenograft tumor growth in SiHa cells. Data represent s mean ± SEM (*n* = 5 mice per group, 2-way ANOVA). (**N**) Representative mIF image of a C3 sample stained for CDH13 (C1, green), TP53BP1 (C2, blue), NNMT (C3, yellow), and HSPB1 (C4, red). (**O**) MRI images of immune patient 9 before (left) and after (right) treatment. Scale bars: 10 cm. (**P**) Response to PD-1/CTLA-4 blockade treatment in the C3 subgroup patients and non-C3 subgroup patients. *P < 0.05, **P < 0.01, ***P < 0.001.

Having established the clinical relevance of our subtypes, we next explored subtype-specific therapeutic strategies. Based on RNA-seq–derived gene expression signatures, we classified S12, SiHa, Ca Ski, and ME-180 cell lines into subtypes C1, C2, C3, and C4, respectively (Figure 5A and Supplemental Note 6). These representative cell lines were subsequently used for functional validation of subtype-specific vulnerabilities. Since the defining molecular feature of the C1 subtype is EMT, we hypothesized that metformin, a compound known to inhibit EMT-related transcription factors including *ZEB1*, *SNAIL2*, *TWIST*, and *VIM* (22), might serve as a potential therapeutic agent for this subtype. Treatment of C1-representative S12 cells with increasing concentrations of metformin significantly reduced their migration and invasion capabilities (Figure 5, B and C, and Supplemental Figure 11, A and B).

Given that the C2 subtype exhibited the poorest prognosis and highest proportions of essential mRNAs (14%, 72 of 522) and proteins (24%, 98 of 414; Figure 5, D and E), we sought to identify therapeutic targets among its characteristic molecular features. We first identified 435 proteins with higher expression in C2 relative to other subtypes. To screen the potential candidates, we combined prognostic information and essential gene data (Supplemental Figure 12A): (a) 381 proteins associated with poor patient overall survival; and (b) gene essentiality scores below –0.7 from CRISPR screening data (21, 23) (Figure 5, D and E, and Supplemental Note 7; Methods). These criteria narrowed down the candidates and prioritized *MFAP1* (score: –1.66) and *SF3B5* (essentiality score: –3.21) as C2-specific targets (Figure 5, F and G).

*MFAP1* and *SF3B5*, both key components were involved in mRNA splicing via spliceosome (24–26). To validate the functional relevance of these candidates, we performed CRISPR/Cas9-mediated knockout of *MFAP1*and *SF3B5* in C2-specific SiHa cells (Figure 5, H and K). Colony formation assays revealed a significantly impaired cell clonogenic capacity following the loss of either gene compared with controls (Figure 5, I and L, and Supplemental Figure 11, C and D). Moreover, in xenograft models, knockout of *MFAP1* or *SF3B5* markedly suppressed tumor growth rate and reduced tumor volume relative to scrambled sgRNA-expressing cells (Figure 5, J and M). Splicing analysis revealed that *MFAP1* knockout promoted the inclusion of exon 3 in *RBM4*, resulting in reduced expression of the short transcript (*RBM4-S*). This splicing shift resulted in inhibition of the downstream mTORC1 pathway, as evidenced by reduced phosphorylation of S6K (Supplemental Figure 12, B–F). Thus, *MFAP1* promotes tumor progression by orchestrating a critical spliceosome-dependent oncogenic program that modulates the RBM4-mTORC1 axis.

C3 was characterized by a heightened immune response, with substantial T cell infiltration and elevated expression of immune checkpoints. To evaluate its predictive value for immunotherapy, we assessed a separate cohort of patients with CC (*n* = 21) who received PD-1/CTLA-4 blockade (Supplemental Table 4). Using our 4-protein classifier, 10 of the 21 pretreatment samples were classified as C3 subtype, and 11 as non-C3 (Figure 5N). Based on RECIST criteria (v1.1) (27), patients were categorized as responders (*n* = 11) or nonresponders (*n* = 10) (Figure 5O). Notably, 90% of C3 patients (9 of 10) responded to treatment, compared to only 18% (2 of 11) in the non-C3 group (*P* = 0.002, Fisher’s exact test; Figure 5P). This noticeable difference underscores the utility of both the four-protein classifier and C3 subtyping in predicting immunotherapy responsiveness.

Subtype C4 was marked by epithelial differentiation and elevated keratin expression. Among these, KRT16 (28) emerged as a potential therapeutic target for C4-subtype patients. To assess its functional relevance, we performed CRISPR/Cas9-mediated knockout of KRT16 in C4-specific ME-180 cells (Figure 5Q). In vitro analyses revealed that KRT16 deletion substantially reduced colony formation (Figure 5R). This effect was further confirmed in vivo, as KRT16-knockout xenograft models exhibited impaired growth compared with controls (Figure 5S). IHC analysis of C4 tumors (Supplemental Figure 13) demonstrated a positive correlation between KRT16 expression and phosphorylation of SRC, implicating KRT16 may promote tumorigenesis through modulation of SRC-mediated signaling pathways.

Collectively, we validated subtype-specific therapeutic targets and the 4-protein classifier in both preclinical models and clinical cohorts, advancing the feasibility of subtype-guided therapeutic strategies for individual patients with CC.

### Global phosphoproteomic profiling identifies subtype-specific kinase activities and therapeutic targets.

**Figure 6:**
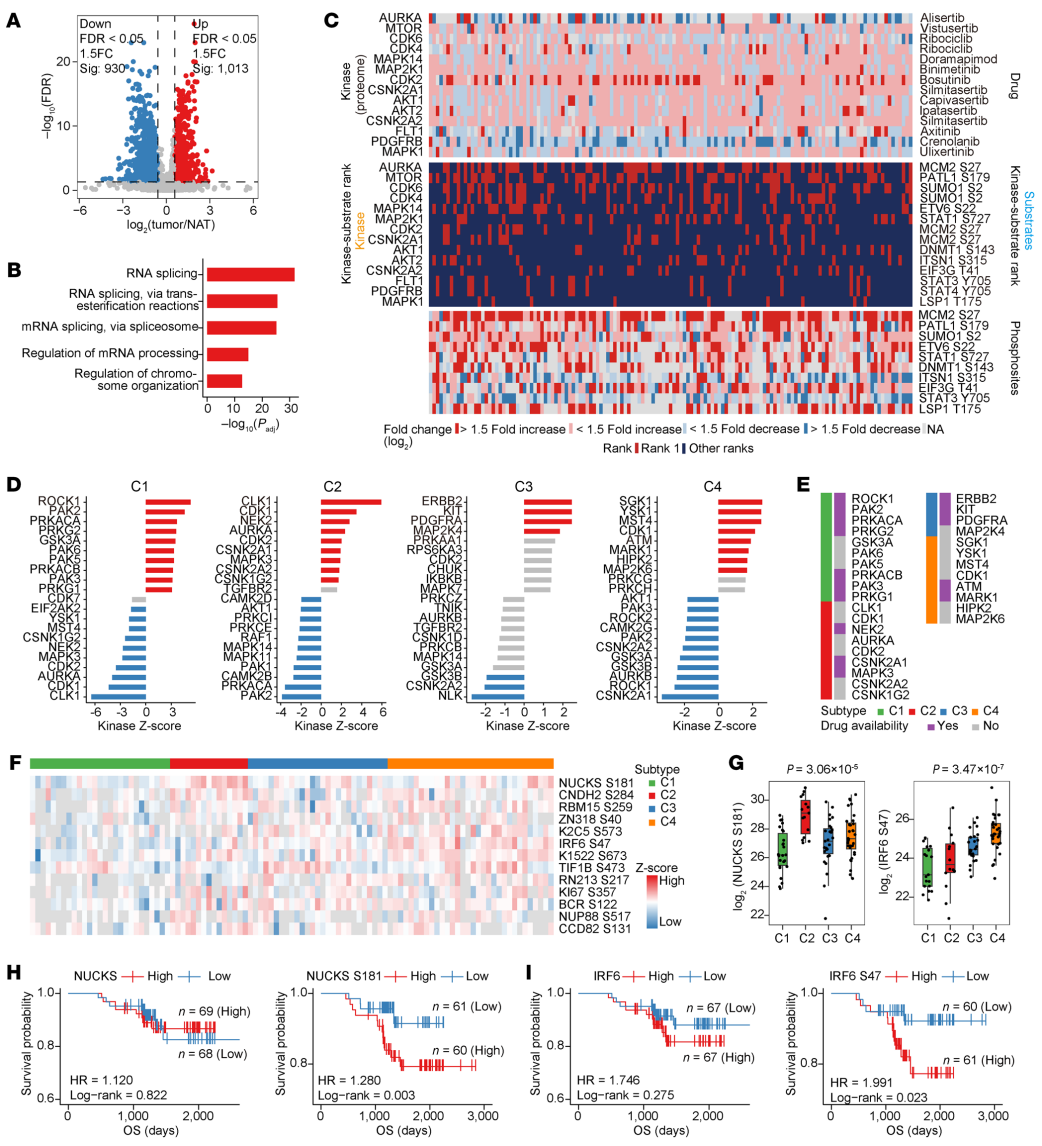
Phosphoproteomics landscape of CC. (**A**) Volcano plot illustrating differentially expressed phosphosites between tumors and the paired NATs (Benjamini-Hochberg adjusted *P* < 0.05, fold change > 1.5, paired modified *t* test). (**B**) Representative enriched GO-BP terms for phosphosites upregulated in tumors compared with paired NATs. (**C**) Ranked phospho-substrate events (middle) for kinases with FDA-approved drugs, showing fold change at global- (top) and phospho- (bottom) levels for kinases and substrates, respectively. (**D**) Evaluation of kinase activities by KSEA using abundance data of phosphosites specifically expressed in each subtype (Benjamini-Hochberg adjusted *P* < 0.05). Red indicates activated kinases, and blue indicates inhibited kinases. (**E**) Heatmap illustrating the drug availability of activated kinases in all subtypes. (**F**) Heatmap of the abundance of 13 prognostic phosphosites upregulated in tumors across all 101 patients. (**G**) Box plot showing NUCKS S181 (left) and IRF6 S47 (right) abundances across 4 subtypes (1-way ANOVA test). Centers indicate the medians, the upper and lower boundaries of the boxes indicate the 75th and 25th percentile, whiskers extend to 1.5× IQR. (**H**) Kaplan-Meier curves for OS of patients based on median value of NUCKS (left) and NUCKS S181 (right) abundances (Log-rank test). (**I**) Kaplan-Meier curves for OS of patients based on median value of IRF6 (left) and IRF6 S47 (right) abundances (Log-rank test).

Having characterized distinct proteomic signatures across the 4 CC subtypes, we next performed global phosphoproteomic analysis to delineate subtype-specific kinase activities and their regulatory roles in shaping the proteomic landscape of each subtype. By assessing phosphosite abundance across 139 tumors and NATs, we quantified 8,259 phosphosites present in at least 20% of samples. Among these, 1,013 phosphosites were significant upregulated, while 930 were significant downregulated in tumors compared to NATs (FDR < 0.05, fold change > 1.5, paired modified *t* test; Figure 6A). The phosphosites with elevated abundance in CC showed enrichment in RNA processing pathways (Figure 6B and Supplemental Table 5).

We subsequently analyzed kinase-substrate interactions, focusing on kinases with available drugs and quantifiable protein expression in at least 50% of patients. For each kinase, the phosphosite with the highest abundance in each tumor/NAT pair was identified as the rank-1 substrate. This analysis identified 14 rank-1 kinase–substrate pairs, representing key phosphorylation events potentially driving CC progression (Figure 6C and Supplemental Table 5).

To explore subtype-specific signaling pathways, we performed kinase-substrate enrichment analysis (KSEA), which revealed distinct kinase activation across subtype (Figure 6D). The p21-activated kinase (PAK) family (PAK2/3/5/6) was specifically activated in the C1 subtype. Similarly, ROCK1, a regulator of EMT (29), showed elevated activity in C1 but was suppressed in C4. The C2 subtype exhibited activation of CLK1, NEK2, AURKA, MAPK3, and CDK1/2, with CLK1 being strongly activated in C2 and inhibited in C1. Receptor tyrosine kinases such as ERBB2, KIT, and PDGFRA were enriched in C3, whereas SGK1, YSK1, and MST4 dominated in C4. By cross-referencing KSEA results with DrugBank (30), we identified several subtype-specific kinases targetable by FDA-approved inhibitors or compounds in clinical development (Figure 6E).

We next validated the therapeutic potential of kinase inhibitors identified in KSEA analysis. In S12 cells, treatment with Fasudil suppressed phosphorylation of MYPT1 (the downstream of ROCK1) (Supplemental Figure 14, A and B). SiHa cells exhibited marked sensitivity to Ulixertinib (MAPK3 inhibitor), as treatment potently suppressed phosphorylation of its downstream effectors C-MYC and RSK (Supplemental Figure 14, C and D). Similarly, ME-180 cells responded to Fostamatinib (MARK1/NEK2 inhibitor), which correspondingly reduced phosphorylation of MARK and its substrate FLT3 (Supplemental Figure 14, E and F). These findings confirm the functional significance of subtype-specific kinase activation and underscore the potential of molecular subtyping in guiding precision therapy for CC.

Differentially phosphorylated proteins can also serve as prognostic markers. We identified 13 significantly upregulated phosphosites associated with prognostic relevance (*P* < 0.05, Log-rank test; Figure 6F and Supplemental Table 5). Among them, NUCKS S181, a phosphosite implicated in DNA replication, transcriptional regulation, and chromatin condensation, exhibited the strongest association with poor prognosis (*P* = 0.003, Log-rank test; Figure 6H). Notably, elevated phosphorylation of NUCKS S181 were predominantly observed in the C2 subtype (*P* = 3.06 × 10^–5^, ANOVA test; Figure 6G). Similarly, IRF6 S47, a site involved in epidermal development (31), showed elevated abundance in the C4 subtype and was associated with poor OS (*P* = 0.023, Log-rank test; Figure 6I). Interestingly, although both phosphosites were prognostically relevant, the total protein abundances of NUCKS and IRF6 were not robustly associated with patient outcomes (Figure 6, H and I). These findings highlight the added prognostic value of phosphoproteomic data and its ability to reveal regulatory events beyond conventional proteomic profiling.

### Proteogenomic profiling reveals subtype-associated distribution of ecDNAs.

**Figure 7:**
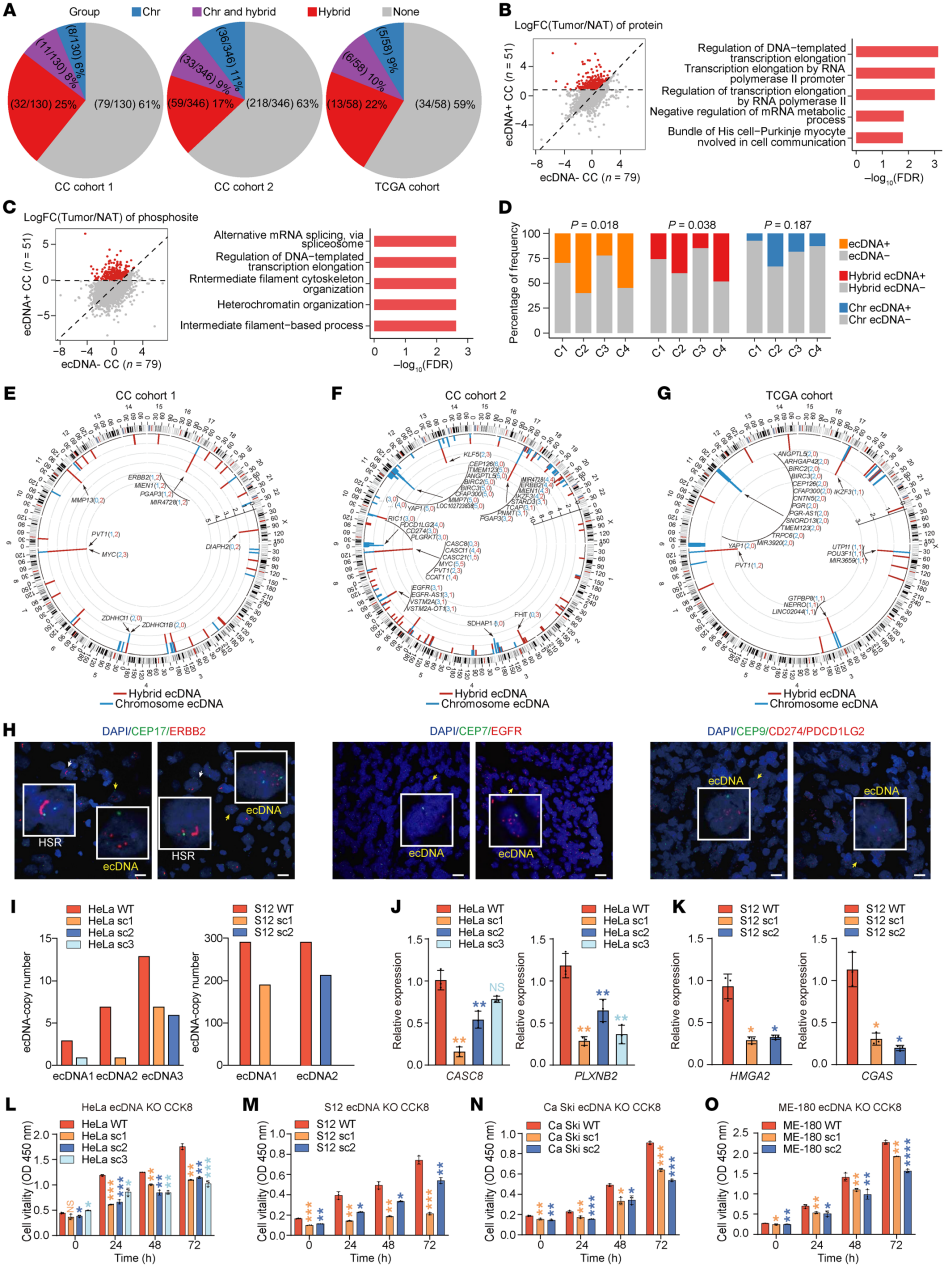
ecDNA analysis in CC. (**A**) Proportion of samples harbored chromosome-only, chromosome and hybrid, or hybrid-only ecDNAs in CC cohort 1 (left), CC cohort 2 (median), and the TCGA cohort (right). (**B**) Fold changes (tumor/NAT) of proteins in ecDNA+ samples versus ecDNA– samples (left). Red dots indicate proteins with > 1.5-fold increase in tumors compared to NATs and higher expression in ecDNA+ compared with ecDNA– cases. Enriched GO terms for these proteins are shown (right). (**C**) Fold changes (tumor/NAT) of phosphosite levels in ecDNA+ and ecDNA– samples (left). Red points denote phosphosites with > 1.5-fold increase in tumors and greater change in ecDNA+ cases. Enriched GO terms for these phosphosite corresponding proteins are shown (right). (**D**) Distribution of samples harbored ecDNAs, hybrid ecDNAs, and chromosome ecDNAs across subtypes in CC cohort 1. The differences of distribution across subtypes were tested by Fisher test. (**E**–**G**) Human genomic distribution of recurrently detected genes located on hybrid or chromosome ecDNAs in CC cohort 1 (**E**), CC cohort 2 (**F**), and the TCGA cohort (**G**). (**H**) Representative FISH images of ecDNA carrying *ERBB2*, *EGFR*, and *CD274/PDCD1LG2* in FFPE samples from CC cohort 1 and CC cohort 2. Scale bar: 10 μm. (**I**) The ecDNA copy numbers in 3 ecDNA knockdown HeLa monoclones (left) and 2 ecDNA knockdown S12 monoclones (right). (**J** and **K**) The RT–qPCR analysis of relative expression levels of cis-genes (*CASC8* and *HMGA2*) and trans-genes (*PLXNB2* and *CGAS*) in 3 HeLa monoclones (**J**) and 2 S12 monoclones (**K**). (**L**–**O**) The CCK8 proliferation assay of hybrid ecDNA knockout mono-clones of HeLa (**L**), S12 (**M**), Ca Ski (**N**), and ME-180 (**O**). Data represent means ± SEM (*n* = 3 biological replicates, 2-sided Student’s *t* test), *P < 0.05, **P < 0.01, ***P < 0.001.

Because extrachromosomal DNA (ecDNA) has emerged as a prevalent mechanism of oncogenesis in human cancers (32, 33), we next sought to characterize the ecDNA landscape and its association with molecular subtypes in CC. Based on WGS data and the AmpliconArchitect algorithm, we identified ecDNAs in 39% (51 of 130) of patients from CC cohort 1 (Figure 7A), a proportion comparable to the 24%–60% range reported in other cancers (34). Notably, hybrid human–viral ecDNAs (35) were detected in 84% (43 of 51) of ecDNA+ samples (Figure 7A). In consistency, the proportion of ecDNA+ tumors was 37% (128 of 346) in CC cohort 2 and 41% (24 of 58) in the TCGA cohort (Figure 7A). Hybrid ecDNAs were similarly prevalent: 72% (92 of 128) in CC cohort 2 and 79% (19 of 24) in the TCGA cohort (Figure 7A). Further analysis of CC cohort 1 revealed that ecDNA+ tumors exhibited elevated expression of proteins enriched in RNA export, transcriptional elongation, and alternative splicing pathways, compared with both NATs and ecDNA– samples (Figure 7B, Supplemental Figure 15A, and Supplemental Table 6). The pattern was validated in the phosphoproteome, where phosphosites with elevated abundance in ecDNA+ samples also showed enrichment in RNA processing pathways (Figure 7C, Supplemental Figure 15B, and Supplemental Table 6). Further analysis revealed that ecDNA+ tumors exhibited significantly higher chromosomal instability (CIN) scores compared with ecDNA– cases (*P* < 0.001), suggesting that this RNA processing dysregulation is more likely attributable to generalized genomic instability (Supplemental Figure 15, C and D).

We next investigated the relationship between ecDNA and molecular subtypes and revealed that subtypes C2 and C4 contained higher proportions of ecDNA+ samples compared with subtypes C1 and C3 (*P* = 0.018; Figure 7D). Notably, hybrid ecDNA was also most abundant in C2 and C4, with C4 exhibiting the highest prevalence (*P* = 0.038; Figure 7D). In contrast, the distribution of chromosome ecDNA showed no difference among the 4 subtypes (*P* = 0.187; Figure 7D).

Genes located on ecDNA were nonrandomly distributed, with recurrent hotspots at 17q12, 8q24.21, and 11q22.1/11q22.2 (Figure 7, E–G), aligning with identified focal gains (Figure 2B). Eleven hotspot genes were shared across all 3 cohorts (Supplemental Figure 15E). To identify putative tumor-promoting candidates, we focused on genes enriched in ecDNAs with high copy numbers (CN), including *PGAP3* (CN = 274.92) and *ERBB2* (CN = 235.59) in CC cohort 1, *CCAT1* (CN = 617.37) and *EGFR* (CN = 343.07) in CC cohort 2, and *PVT1* (CN = 111.29) and *MIR1206* (CN = 55.73) in the TCGA cohort (Supplemental Figure 15F).

To experimentally confirm ecDNAs predicted by WGS, we examined amplification of *ERBB2*, *EGFR*, and *CD274*/*PDCD1LG2* in formalin-fixed, paraffin-embedded (FFPE) slides using fluorescence in situ hybridization probes that target these key genes (Figure 7H). Most ecDNA+ samples exhibited amplification in the form of ecDNA or a combination of ecDNA and homogeneously staining region patterns. CRISPR/Cas9 targeting of HPV integration regions in HeLa and S12 cells led to a marked reduction in ecDNA copy numbers (Figure 7I). RT-qPCR analysis revealed that ecDNA depletion led to reduced expression of its regulated genes (*CASC8* and *PLXNB2* in HeLa, *CGAS* and *HMGA2* in S12) (Figure 7, J and K). Cell counting kit-8 (CCK8)‌ assays further demonstrated pronounced inhibition of cell proliferation in ecDNA–depleted monoclones compared to WT cells, confirming the oncogenic role of ecDNA in driving transcriptional dysregulation and tumor progression (Figure 7, L and M). Similar results were observed in additional CC cell lines, Ca Ski and ME-180, where CRISPR/Cas9 targeting of hybrid ecDNAs also impaired proliferation (Figure 7, N and O). To assess the specificity of this effect, we performed off-target analysis via amplicon sequencing. The proportion of these off-target effects was minimal compared with the on-target effects (Supplemental Figure 15G).

### HPV integration drives human-HPV fusion peptide production.

**Figure 8:**
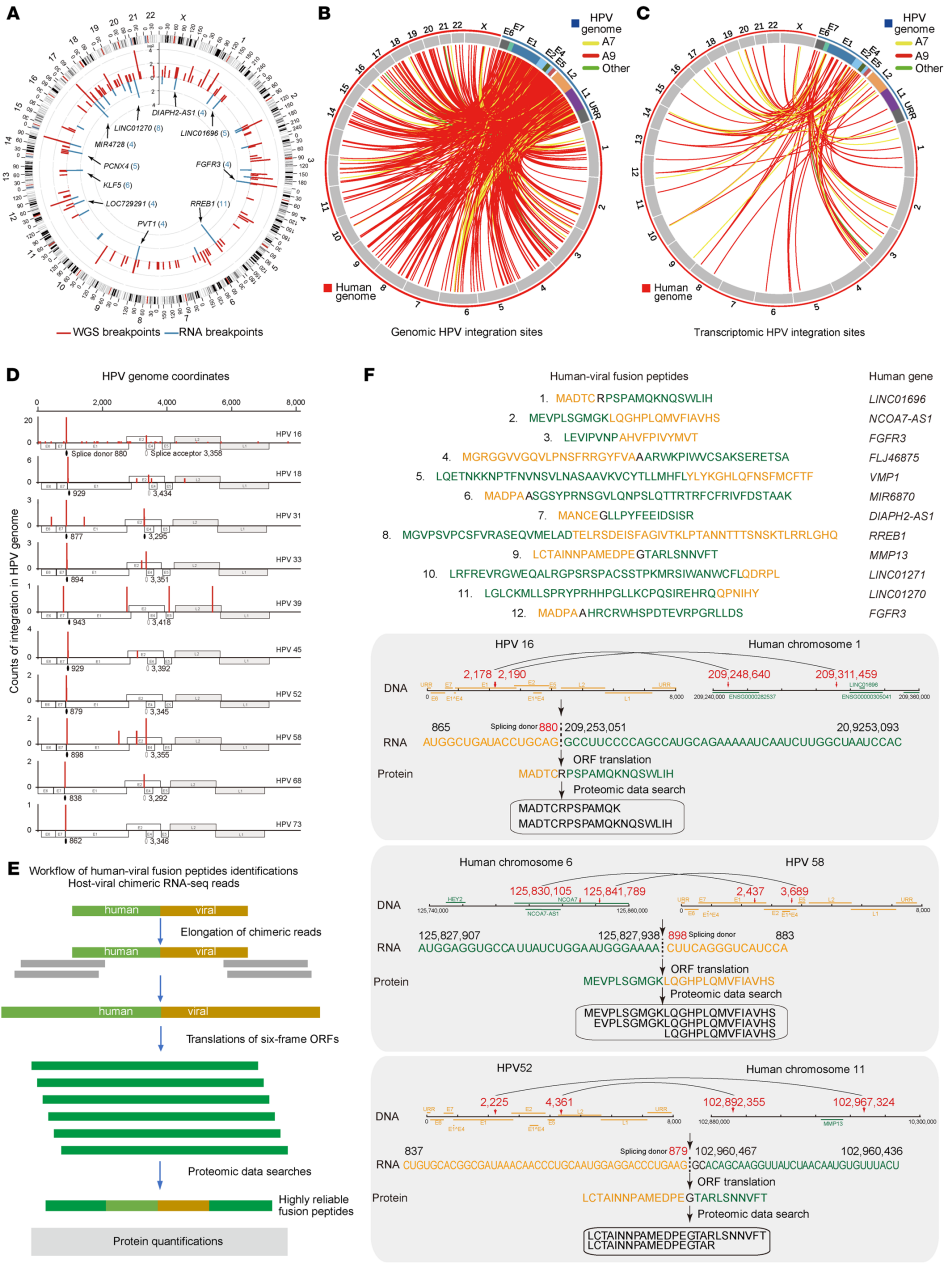
HPV integration and human-viral fusion peptides. (**A**) Distribution of identified integration breakpoints in the human genome. The 10 most frequently integrated genes at RNA level are labeled, number in the brackets represent the integration frequency. (**B** and **C**) Joint Circos plot showing genomic (**B**) and transcriptomic (**C**) integration breakpoints from the HPV genome to the human genome. (**D**) Distribution of integration breakpoints across the HPV genome. The ordinates represent the number of integration events. (**E**) Workflow for the identification of human-viral fusion peptides. (**F**) The 12 peptides derived from the predicted open reading frames (ORF) with positive matches in proteomic searches (top). Detailed information for peptides no.1, 2, and 9 are showed at the bottom.

While our previous study established HPV integration in hybrid ecDNA biogenesis at the genomic level (33), its downstream impact on the transcriptome and proteome remains unexplored. We therefore systematically characterized HPV integration events to delineate their functional consequences (Figure 8A and Supplemental Table 7). Genomic analysis revealed 662 HPV integration events across 130 patients (Figure 8B, Supplemental Figure 16, A and B, Supplemental Note 8, and Supplemental Table 7). In parallel, RNA-seq identified 151 human-viral fusion breakpoints in 55 of 101 patients (Figure 8C and Supplemental Table 7). Among these, the majority of mRNA integration events (98 of 151) originated in the HPV E1 region, followed by 36 events at the E2, and 17 events at the E6/E7/L1/L2/URR loci (Figure 8C and Supplemental Table 7). Importantly, 70% (61 of 87) of transcript-level integration events were located proximity to canonical HPV16 alternative splicing sites, including splice donor 880 (SD880) within E1 and splice acceptor 3358 (SA3358) within E4 (Figure 8D and Supplemental Table 7). These findings suggested a potential association between HPV integration and alternative splicing regulation.

To further investigate the posttranscriptional consequences of HPV integration, chimeric RNA reads were used as anchors to reconstruct fusion transcripts. The resulting transcripts were translated into fusion peptides, which were subsequently matched against proteomic datasets (Figure 8E, Supplemental Figure 16C, and Supplemental Note 8). Through these integrated analyses, we identified 12 human-viral fusion peptides across 11 samples (Supplemental Figure 17). For each fusion peptide, the corresponding genomic breakpoints (within 100 kb of mRNA junctions), reconstructed RNA reads, and the peptide sequences were illustrated in Figure 8F and Supplemental Figure 18. Interestingly, 58.3% (7 of 12) of the fusion peptides originated from noncoding genes, including *LINC01696*, *FLJ46875*, *MIR4728*, and *LINC01270*, whereas the remaining peptides derived from protein-coding genes, including *FGFR3*, *VMP1*, *RREB1*, and *MMP13* (Supplemental Table 7). Even within protein-coding genes, most breakpoints were localized in noncoding regions (Supplemental Table 7). By annotating the breakpoints on the viral genome, we found that 10 out of the 12 fusion peptides were located at or near alternative splicing sites, including SD880 at E1 and SA3358 at E4 (Figure 8D and Supplemental Table 7). Taken together, these data indicated that HPV integration reshapes host transcriptional output and promotes the generation of human-viral fusion peptides.

We then examined whether the 11 samples containing fusion peptides were enriched for hybrid ecDNA. Notably, in 7 of the 11 samples, we observed the existence of hybrid human-HPV ecDNA. Furthermore, in 6 of these 7 samples, the fusion peptide–associated genomic breakpoints were concordant with the human-HPV breakpoints on hybrid ecDNA (Figure 8F). Together, these results supported a potential link between ecDNA and fusion peptide generation in CC.

## Discussion

Advances in genomic research over the past decade have strikingly enhanced our understanding of CC (5–7), yet the lack of comprehensive data on the global proteogenome and phosphoproteome has hindered the development of widely applicable stratification criteria and actionable therapeutic strategies. Our study addresses this gap by presenting the first genome-wide proteogenomic landscape of CC and extends the dimensions of the genomic research, providing a valuable resource for further investigations.

Our multi-omics study reveals 4 distinct molecular subtypes (C1–C4) that both align with and extend beyond current histology subtype and TCGA subtype (5) (Supplemental Note 5 and Supplemental Figure 9), providing biological insights particularly in the C2 and C3 subtypes. While C1 corresponds with EMT signatures and C4 to PI3K–AKT cluster (keratin high), the identification of C2 as a highly proliferative subtype with the poorest clinical outcomes challenges the traditional TCGA paradigm where the EMT subtype was considered of worst prognosis. The discovery of C3 as an immune-enriched subtype, with high response rate to PD-1/CTLA-4 blockade, represents a marked advancement in understanding CC biology and demonstrates immediate therapeutic implications. Our proteogenomic data were further stratified by histopathological subtypes to delineate the differences between squamous cell carcinoma and adenocarcinoma (Supplemental Note 9 and Supplemental Figure 19).

Recent advances in single-cell transcriptomics offer complementary insights into molecular subtypes and therapeutic targets by comprehensively analyzing cellular intrinsic and microenvironmental phenotypes (36). However, its clinical application still faces obstacles due to high sample requirements, complex processing procedures, and substantial costs (37). In contrast, our 4-protein classifier derived from bulk multi-omics data offers an immediate translational utility that enables precise patient stratification and targeted therapeutic selection, bridging the gap between molecular profiling and actionable clinical decisions in CC.

Further, we performed the first comprehensive profiling of ecDNA in 534 cases from three CC cohorts (Figure 7), since ecDNA is increasingly recognized for its role in promoting tumorigenesis and drug resistance (34). We unveiled a nonrandom distribution of genes on ecDNA, indicating a selective mechanism governing their formation and prevalence. Proteogenomic analysis revealed pronounced alterations in protein activity and signaling pathways specific to ecDNA. By identifying and targeting genes associated with ecDNA, we may develop more precise therapeutic strategies through targeted drug therapy or CRISPR-Cas9 systems to modify/eliminate ecDNA (34, 38).

Intriguingly, by integrating matched genomic and proteomic data, our study identified the first 12 proteomics-supported human-viral fusion peptides in CC (Figure 8 and Supplemental Figure 17). These fusion peptides raise interesting questions regarding their molecular form, functional role in oncogenesis, and potential as diagnostic biomarkers/immunotherapeutic targets. Future studies should evaluate peptide-specific T-cell responses (IFN-γ ELISpot and flow cytometry) and antigen-dependent cytotoxicity (in vitro T cell–mediated killing assays) (39, 40).

Limitations of our study warrant careful consideration. First, the proteomic analysis compared epithelial-derived tumor tissue against predominantly mesenchymal normal adjacent tissue. This tissue origin mismatch suggested that NAT may not serve as a perfect baseline for comparison (41). Second, as our study focused on treatment-naive primary tumors, the molecular subtypes, kinase activities and ecDNA prevalence identified here may shift in metastatic/recurrent disease (42, 43). Future studies should validate these findings using appropriately epithelial-matched normal cervical controls and extend analyses to metastatic, recurrent, and posttreatment cohorts to determine how molecular subtypes, kinase activities, and ecDNA prevalence evolve over disease progression and therapy.

Altogether, this comprehensive proteogenomic study markedly broadens our understanding of CC biology beyond what genomic analyses alone can reveal, by elucidating more distinct cancer subtypes. These subtypes hold the promise of guiding the development of precision therapeutics tailored to specific patients. We are optimistic that our findings will not only pave the way for more effective clinical treatments for CC but also serve as a valuable resource for both basic and clinical research communities.

## Methods

### Sex as a biological variable.

Our study exclusively examined female humans and female mice because the disease modeled is only relevant in females.

### Specimen acquisition and preparation.

The biospecimens for this study were prospectively collected from newly diagnosed patients with CC undergoing surgical resection at Wuhan Central Hospital and Tongji Hospital between 2014 and 2019. For CC cohort 1 (*n* = 139), primary tumor tissue, matched adjacent normal tissue, and peripheral blood samples were collected before treatment following standardized protocols (44). Multi-omics profiling was performed on these samples, including WGS (*n* = 130), RNA-seq (*n* = 101), and global proteomic and phosphoproteomic analyses (*n* = 139). Detailed clinical data were provided in Supplemental Table 1.

### DNA extraction and WGS.

Genomic DNA was extracted from tumors and matched peripheral blood samples using the QIAamp Fast DNA Kit (Qiagen). A total of 0.5 μg of high-quality DNA per sample was processed with the TruSeq Nano DNA HT Kit (Illumina). Paired-end sequencing (2 × 150 bp) was conducted on the Illumina HiSeq X platform.

### Somatic variant calling.

Clean reads were aligned to the human reference genome GRCh38 via BWA MEM (v.0.7.17) (45) with default settings. Somatic mutations (SNVs and indels) were identified using the GATK (v.4.0) best-practice pipeline. High-confidence mutations were annotated with Funcotator (v.1.7.20200521s) for genomic features.

### Whole-genome doubling, chromothripsis events, and clonal composition analysis.

Whole-genome doubling (WGD) status was assessed as previously described (46). Major integer DNA copy numbers were inferred using FACETS (v.0.3.9) (47) with a critical value parameter (cval) of 150. Chromothripsis events were analyzed using ShatterSeek (48) (v.0.4). Tumor clonal compositions were inferred with the PhyloWGS algorithm (49).

### SCNA analysis.

SCNA analysis was performed using the GATK somatic CNA Best Practices pipeline (v.4.0). Segment-level ratios were Log_2_-transformed, and significant SCNAs were identified with GISTIC2.0 (v.2.0.23) (Q-value < 0.25). Copy number alterations were defined as gain/loss (Log_2_ ratio ± 0.1) or amplification/deletion (Log_2_ ratio ± 0.8) (19).

### RNA extraction and RNA-seq.

Total RNA was extracted from fresh frozen tissues using TRIzol reagent (Invitrogen). Sequencing was performed on DNBSEQ-T7 platform (MGI), generating 150 bp paired-end reads.

### SCNA-driven cis and trans effects.

SCNA *cis/trans* effects on mRNA and protein were analyzed via Spearman’s correlation (multiOmicsViz R package v.3.18).

### Protein extraction, digestion, and phosphorylated peptide enrichment.

Protein extraction, digestion, and phosphorylated peptide enrichment were performed following the protocol described by Jiang et al (47).

### LC-MS/MS of peptide mixture.

Shotgun proteomics was performed following the protocol described by Jiang et al (47).

### QC of the mass spectrometry platform.

HEK293T cell lysate served as the mass spectrometry quality control (QC) standard, measured every 3 days. Pairwise Spearman’s correlations across QC runs were computed in R and shown in Supplemental Figure 2, A and B.

### Database searching of MS/MS data.

MS/MS data were analyzed using Spectronaut (v.18.0.230605.50606) against the human UniProt database. Trypsin digestion employed up to 2 mis-cleavages for 7–52 amino acid peptides. Fixed modification: cysteine carbamido methylation; variable modifications: protein N-terminus acetylation and methionine oxidation. Peptide-spectrum matches, and protein identifications were filtered at < 1% FDR. Phospho-proteome data were searched via MaxQuant (v.2.0.3.1, RRID: SCR_014485) (48), targeting phosphorylation of serine, threonine, and tyrosine. Mass tolerances were 20 ppm (first search) and 4.5 ppm (main search). Phosphorylation sites with localization probability > 0.75 were considered reliable. Normalized intensities demonstrated successful QC with unimodal distributions across samples (Supplemental Figure 2, C and D). PCA of proteomic profiles revealed no significant batch effects (Supplemental Figure 2, E and F).

### Phosphoproteomic data analysis.

Median normalization was applied, and tumor/NAT fold changes were calculated to identify tumor-elevated phosphosites (> 1.5-fold increase) (18). Kinase substrate ranking was based on high-fold-change prevalence across tumors. Kinase activities were estimated using KSEA (49).

### Subtype clustering.

Tumor subtyping was performed via unsupervised clustering (50) (R package CancerSubtype v.1.28.0) using transcriptome and proteome data. The Execute SNF-CC method integrated SNF and CC. Features were selected by Median Absolute Deviation (MAD), prioritizing top 1,500 MAD mRNAs and 1,000 MAD proteins. Optimal cluster count (k = 2–5) was determined. Subtype-specific mRNAs and proteins required > 1.5-fold upregulation in a subtype versus others (adjusted *P* < 0.01, ANOVA test).

### EMT score.

The EMT score integrated transcripts per million (TPM) from 6 mesenchymal markers (*VIM*, *SNAI2*, *ZEB2*, *FN1*, *MMP2*, *AGER*) and 6 epithelial markers (*CDH1*, *CDH3*, *CLDN4*, *EPCAM*, *ST14*, *MAL2*) (51). The TPM values for each marker was z-scored. The EMT score was computed as the sum of mesenchymal z-scores minus the sum of epithelial z-scores.

### Proliferation score.

The proliferation score was computed as previously described (52), using 654 cell cycle-regulated genes (of 720 identified by Whitfield et al.) detected in our data (53). TPM values were z-scored and summed to derive the proliferation score.

### Immune score and immune cell type composition.

The Immune scores and stromal scores were calculated via the ESTIMATE (54) algorithm using TPM data. Immune cell abundances in CC samples were quantified using CIBERSORTx (55), xCell (56), and MCP-counter (57), all applied to TPM data.

### Epithelial score.

TPM data of a total of 200 epithelial genes (58) were scaled and the summation of scaled values represented the epithelial score.

### Identification of subtype-specific markers using LASSO.

We used LASSO regression with area under the receiver operating characteristic curve (AUC) evaluation to identify subtype-specific marker proteins. LASSO regression was performed (glmnet package) with optimal lambda selected via 10-fold cross-validation. Proteins with non-zero coefficients were retained, and their discriminative power was assessed using AUC (pROC package).

### Alternative splicing analyses in SiHa and HeLa cells.

Differentially spliced exons and introns were identified using rMATS (59), based on RNA-seq data. Significance criteria were established as FDR ≤ 0.01 and |ΔPSI| ≥ 0.05.

### Identification of ecDNA.

AmpliconArchitect (v.1.3.r4) (60) identified ecDNA structures using HPV reference sequences and copy number-aberrant regions from WGS data as previously reported (33).

### HPV integration analysis.

HPV integration sites and human-HPV fusion reads were identified from WGS/RNA-seq data using VIPA (61) and SurVirus (62). Peptides from these ORFs were MS/MS-searched, with only those spanning human-HPV junctions and meeting stringent quality thresholds (Q < 0.01) retained for further analysis.

### Cell lines.

Human CC cell lines including SiHa, HeLa, HEK293T, Ca Ski and ME-180 were purchased from American Type Culture Collection (ATCC). S12 cells were obtained as a gift from K. Raj and M. Stanley (63). S12 cells were cultured in F12 medium/DMEM (3:1) with 5% fetal bovine serum (FBS). SiHa, HeLa, HEK293T cells were cultured in DMEM with 10% FBS. Ca Ski cells were cultured in RPMI 1640 medium with 10% FBS. ME-180 cells were cultured in McCoy’s 5A medium with 10% FBS.

### CRISPR-Cas9 knockout.

*RABIF*, *SCAMP3*, *MFAP1*, *SF3B5* and *KRT16* depletion were conducted using CRISPR-Cas9 gene editing technology. The sgRNA sequences are as follows: Scramble (Scr) (GTGTGCGTATGAAGCAGTG-3’), RABIF (sg1 5’-AGCCGAGGGCCGAAACCGGA-3’; sg2 5’-TCGGCTGACACTAACTCGCT-3’), SCAMP3 (sg1 5’- CATCCAGAGGTAGTACATGG-3’; sg2 5’- GTATCCACCATGTACTACCTC-3’), MFAP1 (sg1 5’-AGCGTTATGTGTCCGGAAAA-3’; sg2 5’-AAGGTAAAGCGTTATGTGTC-3’), SF3B5 (sg1 5’-TGGCCCGTGCCGATGTACT-3’; sg2 5’-CGAGTCCGCTTCAACTTGA-3’), KRT16 (sg1 5’- AGAAGCGAGAGGAGACAGAC-3’; sg2 5’- GCTCATCCAACACCCGGCGC-3’).

### Western blotting.

Proteins were lysed in urea buffer with protease/phosphatase inhibitors, and concentrations determined by Bradford assay. Samples (10–20 μg) were resolved by SDS-PAGE, transferred, and probed with primary antibodies. HRP-conjugated secondary antibodies were used, with signal detection via chemiluminescent imaging. GAPDH and β-tubulin were used as internal loading controls. Detailed for all the antibodies were provided in Supplemental Table 8.

### Glucose uptake assay.

Glucose uptake was measured in SiHa cells using the ab134955 assay kit (Abcam) as previously reported (20).

### Fluorescent in situ hybridization (FISH).

DNA FISH was performed using probes for *ERBB2/CEP17*, *CD274/PDCD1LG2/CEP9* (Wuhan HealthCare Biotechnology), and *EGFR/CEP7* (Guangzhou Exon Biotechnology), as previously reported (33).

### Knockdown of hybrid ecDNA.

HPV integration regions on ecDNAs were targeted and knocked down using CRISPR-Cas9 gene editing technology, as previously described (33).

### IHC staining analysis.

A CC tissue microarray (RH-J-7-C14, 102 cores) was used to assess the expressions of RABIF and SCAMP3. Expression of CD4, CD8, CD68, and α-SMA were tested in FFPE tissue sections of CC to validate the xCell analysis of tumor microenvironments. Two blinded pathologists independently scored the IHC slides using established criteria.

### Multiplexed immunostaining.

A CC tissue microarray (RH-J-7-D11, 102 ores) was subjected to multiplex immunostaining using the Opal Polaris 7-Color Manual IHC Detection Kit. Primary rabbit monoclonal antibodies targeting TP53BP1, CDH13, NNMT, and HSPB1were sequentially applied. After each round of antibody application, Opal Anti-Ms+Rb HRP and TSA-DIG buffers were used, with microwave treatment between rounds to eliminate nonspecific signals. After DAPI counterstaining and mounting, the slides were scanned using the AKOYA Pheno Imager HT.

### Subcutaneous xenograft models.

Female BALB/c nude mice (4–6 weeks old) obtained from Beijing SPF biotechnology Co. Ltd, were used to assess gene function via subcutaneous xenograft models. Cells (Scr/KO) were suspended in PBS/Matrigel and injected into the flanks of the mice as follows: RABIF (1 × 10^6^ HeLa or 5 × 10^6^ SiHa), SCAMP3 (1 × 10^6^ HeLa or 5 × 10^6^ SiHa), MFAP1 (5 × 10^5^ SiHa), SF3B5 (5 × 10^6^ SiHa), and KRT16 (1 × 10^7^ ME-180), with 5 mice per group. Tumor volumes were measured every 2–3 days using calipers.

### Statistics.

Quantitative data are presented as mean ± SEM. Continuous variables were compared using 2-sided Student’s *t* tests, Wilcoxon rank-sum tests, or 1-way ANOVA with Tukey’s multiple comparisons test. Categorical data were assessed using χ^2^ or Fisher’s exact tests. Survival (OS and PFS) was estimated using Kaplan-Meier curves and compared by Log-rank tests. Hazard ratios were calculated using Cox proportional hazards models. *P* values for multi-omics and multiple comparisons were adjusted using the Benjamini-Hochberg method. *P* < 0.05 was considered statistically significant. Statistical analyses were performed using R (v4.4.2) and GraphPad Prism 9.0.

### Study approval.

The study protocol involved humans and was approved by the Institutional Review Boards of Wuhan Central Hospital and Tongji Hospital, and written informed consent was obtained from all participants. All the mouse studies were performed in compliance with the guidelines of Institutional Animal Care and the Ethics Committee of the Tongji Medical School.

### Data availability.

The genotype data generated by WGS have been deposited in GSA for Human in the National Genomics Data Center (https://ngdc.cncb.ac.cn/gsa-human) under controlled access due to data privacy laws related to patient consent for data sharing with accession number HRA016382. The bulk RNA-Seq data have been deposited in GSA for Human in the National Genomics Data Center under the accession code HRA007904. Raw files of proteome and phosphoproteome datasets have been deposited in iProX database (www.iprox.org) under accession number IPX0006107001 and IPX0006107002, respectively. The values for all data points in graphs are reported in the Supporting Data Values file.

## Author contributions

ZH and XT conceived, designed, and supervised the research. ML, YL, ZW, and SW conducted the bioinformatics analysis. YZ and ML supervised and guided the bioinformatics analysis. JF, LW, ZJ, XZ, ZY, ZW, TF, and YW performed the functional experiments. RT was responsible for CRISPR library and gene editing. ZW, TF, CC, and YW conducted the statistical analysis. XT, ML, ZW, TF, YL, CC, and YW wrote the manuscript. ZH, XT, YZ, and HW offered research guidance. ZH, MY, HW, and YZ revised the manuscript. Author order was determined by the corresponding authors based on overall contribution.

## Funding support

- Noncommunicable Chronic Diseases-National Science and Technology Major Project (2025ZD0544102 to ZH).
- The National Natural Science Foundation of China (82172584 to XT).
- Key Technology R&D Program of Hubei (2024BCB057 to XT, 2025BCB053 to CC).
- National Natural Science Foundation of China (82373260 to HW).
- The “4+X” clinical trial programs of Women’s Hospital, School of Medicine, Zhejiang University (LY2022004 to HW).
- The programs of Zhejiang Traditional Chinese Medicine Innovation Team (CZ2024009 to HW).
- Guangxi Natural Science Foundation (2024GXNSFBA010045 to MY).

## Supplementary Material

Supplemental data

ICMJE disclosure forms

Unedited blot and gel images

Supplemental table 1

Supplemental table 2

Supplemental table 3

Supplemental table 4

Supplemental table 5

Supplemental table 6

Supplemental table 7

Supplemental table 8

Supporting data values

## Version 1 02/10/2026

In-Press Preview

## Version 2 04/01/2026

Electronic publication
