# Supplementary material for: Proteogenomic characterization of cervical cancer identifies molecular subtypes predictive of clinical outcomes and subtype-specific targets
Source: J Clin Invest. 2026 Feb 10;136(7):e199497. doi: 10.1172/JCI199497 (PMC13038215; doi:10.1172/JCI199497)
Supplement: Supplemental data [file jci-136-199497-s292.pdf]

Supplemental materials

Supplemental Notes

Supplemental Note 1. Mutation signatures in CC.

Mutational signatures analysis identified three patterns in CC. SBS96A, prevalent in 47% of cases, best resembled Catalogue of Somatic Mutations in Cancer (COSMIC) signature 2/13 (APOBEC-related) (1) with a cosine similarity of 0.984. SBS96C, observed in 51% of samples, aligned with COSMIC signature 5 (aging-related) with a cosine similarity of 0.940. SBS96B, present in 2% of cases, resembled COSMIC signature 33 (unknown etiology) with a cosine similarity of 0.989 (Supplemental Figure 1, A and B). Consistent with previous reports (2), HPV+ tumors exhibited a significantly higher APOBEC-associated mutational burden compared to HPV- tumors ($P = 0.002$, Fisher's exact test; Supplemental Figure 1C).

Supplemental Note 2. Identification of potential therapeutic targets in CC based on protein overexpression profiles.

Among the 23 proteins with more than a 1.5 $\log_2$ fold change in at least 90% of tumors (Supplemental Figure 3C), TACSTD2, which is overexpressed in various solid tumors, is a well-established target of ADC with promising clinical efficacy in cancers including triple negative breast cancer, small cell lung cancer, and colorectal cancer (3). Additionally, TFRC and TYMP are targeted by FDA-approved drugs. Multiple components of the MCM complex, including MCM2/3/5/6/7, which are crucial for

DNA replication initiation, were also significantly upregulated (4-7). Their overexpression has been associated with poor prognosis in hepatocellular carcinoma and gastric cancer (5-7). These proteins represent valuable candidate therapeutic targets for CC.

Supplemental Note 3. Impacts of *SCAMP3* gain on protein expression and tumor growth.

In tumor samples carrying *SCAMP3* gain, both mRNA and protein expression were concordantly elevated (Supplemental Figure 6, A and B). Experimental assays showed that knockout of *SCAMP3* in SiHa and HeLa cells markedly suppressed colony formation (Supplemental Figure 6, C–F) and attenuated tumor growth in xenograft models (Supplemental Figure 6, G and H). Mechanistic investigation showed that *SCAMP3* depletion reduced growth factor receptor (EGFR) stability and attenuated AKT signaling (Supplemental Figure 6J), indicating that *SCAMP3* supports proliferation by potentiating EGFR signaling. Clinically, high SCAMP3 levels were significantly associated with reduced OS (HR = 2.464, P = 0.02, Log-rank test), and showed a trend toward poorer PFS (HR = 1.803, P = 0.08, Log-rank test; Supplemental Figure 6I and Supplemental Table 3).

Supplemental Note 4. The expression patterns of representative genes to refine the biological identity of each subtype.

Subtype C1 displayed pronounced upregulation of canonical EMT-related transcriptional factors including *TWIST1/2*, *SNAI1*, and *ZEB1/2* (8) (adjusted P value < 0.01, ANOVA test; Supplemental Figure 8). Subtype C2 showed high levels of proliferation markers including TOP2A (9), PCNA (10), and MKI67 (adjusted P value < 0.01, ANOVA test; Supplemental Figure 8). Subtype C3 demonstrated elevated expression of immune stimulators such as *IL2RA*, *CD27*, *CXCL10*, *CD28*, *TNFRSF4*, and *TNFRSF18* (11) (adjusted P value < 0.01, ANOVA test; Supplemental Figure 8). Lastly, subtype C4 was enriched for keratin-related epithelial markers, including KRT16, KRT6A, KRT6B, KRT6C, KRT5, KRT15, SFN, and KRT14 (adjusted P value < 0.01, ANOVA test; Supplemental Figure 8).

Supplemental Note 5. Correlation of CC molecular subtypes with TCGA RPPA clusters.

We constructed an XGBoost classifier using the overlapping mRNA and protein features shared between our in-house cohort and the TCGA-CESC dataset. To derive an optimal and robust model architecture, we incorporated grid search and stratified cross-validation during the training phase, alongside an independent validation process in the testing phase. This optimized classifier was subsequently applied to TCGA-CESC samples for subtype stratification, where each case was assigned to one of the four predefined subtypes (C1–C4). Our classification system showed substantial concordance with previously established TCGA subtypes (Supplemental Figure 9 A–E). Specifically, our C1 (EMT-high) subtype aligned with the TCGA keratin-

low/RPPA–EMT cluster, while our C4 subtype corresponded to the TCGA keratin-high/RPPA–PI3K–AKT cluster. These alignments validated the biological relevance of our classification framework.

Beyond these concordant findings, our analysis also revealed novel insights not captured by the original TCGA classification. The C2 subtype, characterized by proliferative activity and immune-cold features, showed the strongest alignment with the TCGA "Hormone" cluster (13/19 cases, 68%), with the remaining cases distributed across the TCGA EMT (3 cases, 16%) and PI3K-AKT (3 cases, 16%) clusters. Furthermore, we identified a distinct C3 immune-hot subtype with favorable prognosis that was not explicitly defined in the original TCGA classification.

Supplemental Note 6. Identification of subtype-specific cell lines.

To validate our proposed subtype-specific therapeutic strategies, we analyzed RNA-seq data from four cervical cancer cell lines (S12, SiHa, Ca Ski and ME-180), focusing on gene expression patterns representative of the four molecular subtypes. The results revealed distinct subtype-specific profiles: S12 cells showed elevated expression of EMT-associated transcription factors, aligning with the C1 subtype (Figure 5A). SiHa cells demonstrated overexpression of cell cycle regulation genes, corresponding to the C2 subtype (Figure 5A). Ca Ski cells exhibited high expression of immune-related genes, indicative of the C3 subtype, while ME-180 cells demonstrated marked overexpression of keratin proteins, placing them in the C4 subtype (Figure 5A).

Supplemental Note 7. Uncovering subtype-specific genetic vulnerabilities through DepMap essentiality profiling.

To elucidate the functions of subtype-specific genes, we assessed the genetic vulnerabilities of proteins and mRNA uniquely expressed in each subtype, using data from the DepMap project (https://depmap.org/). We calculated the average gene essentiality scores, which indicate tumor cell survival dependencies, across 15 CC cell lines after the knockout of 1,188 genes encoding subtype-specific proteins. Essential proteins were defined as those with gene essentiality scores below -0.7. Notably, 23.67% (98/414) of proteins in the C2 subtype were essential, compared to just 1.30% (6/462) in C1, 1.35% (1/74) in C3, and 8.8% (21/238) in C4 (Figure 5E). The high proportion of essential proteins in C2 suggests a critical survival advantage for tumor cells in this subtype, which may contribute to its poorer prognosis.

Supplemental Note 8. Human–HPV fusion transcripts and fusion peptides, stratified by HPV type.

As shown in Supplemental Table 7B and Supplemental Figure 16, A and B, we identified a total of 151 human-viral fusion transcripts. Of these, 87 (57.6%) were derived from HPV16, and 15 (9.9%) were from HPV18. Integration sites differed between HPV16 and HPV18. The majority of breakpoints in the viral genome were mapped to the early genomic regions in both HPV types. Integration breakpoints distributions differed between HPV16 and HPV18. In both HPV types, most viral breakpoints were located in the early genomic region. Specifically, E1 harbored the

highest proportion of breakpoints (HPV16: 57/87, 65.5%; HPV18: 10/15, 66.7%), followed by E2 (HPV16: 18/87, 20.7%; HPV18: 4/15, 26.7%). In the human genome, chromosome 17 was the most frequent breakpoint location for both HPV16 (14/87, 16.1%) and HPV18 (5/15, 33.3%).

At the peptide level, among 12 detected human–HPV fusion peptides, 5 (41.7%) were derived from HPV16 and only 1 (8.3%) from HPV18 (Supplemental Figure 16C). HPV16-derived fusion peptides comprise five entries, of which four originate from the viral E1^E4 region and one originates from E2; on the human side, these five peptides map to four genes: LINC01696, FLJ46875, FGFR3, and MIR6870 (Supplemental Table 7D). In contrast, only one HPV18-derived fusion peptide is observed, which originates from viral E2 and maps to an intronic breakpoint within the human gene VMP1 on chromosome 17 (Supplemental Table 7D).

Supplemental Note 9. Analysis of the main results stratified by major histopathologic type squamous cell carcinoma and adenocarcinoma.

Genomic profiling

When stratified by histology, squamous cell carcinomas were mainly characterized by mutations in *PIK3CA* (26.5%, 30/113) and *KMT2C* (12.4%, 14/113), followed by *SYNE2* (8.0%, 9/113), *HUWE1* (7.1%, 8/113), *FBXW7* (7.1%, 8/113), *KMT2D* (6.2%, 7/113), *NFE2L2* (5.3%, 6/113), *PTEN* (5.3%, 6/113), *EP300* (4.4%, 5/113) and *KLF5* (3.5%, 4/113). In adenocarcinomas, the most frequent mutations were observed in *PIK3CA* (16.7%, 2/12) and *KLF5* (16.7%, 2/12), followed by *HUWE1* (8.3%, 1/12) and

EP300 (8.3%, 1/12) (Figure 1B).

Squamous cell carcinoma and adenocarcinoma distribution across molecular subtypes

We further observed a strong association between molecular subtype and squamous cell carcinoma and adenocarcinoma histology (Figure 3A). Of note, C4 consisted exclusively of squamous cell carcinoma (100%, 32/32) while C3 was predominantly squamous cell carcinoma (96.2%, 25/26). On the other hand, adenocarcinoma histology was mainly distributed in C1 (54.5%, 6/11) and C2 (36.4%, 4/11) subtypes.

Phosphoproteomic profiling

Based on the kinase Z-score analysis, squamous cell carcinomas and adenocarcinomas exhibited both similarities and differences in their kinase activation patterns. Both cancer types shared upregulation of CLK1, AURKA, PRKDC, CDK1, and CDK2, as well as common downregulation of ROCK1, GSK3A, PRKG2, PDHK3, and ADRBK1. However, squamous cell carcinomas were mainly characterized by upregulation of HIPK family members, NEK2, and CHEK1, along with downregulation of CDK5, ROCK2, PRKG1, DAPK1, and LIMK2 (Supplemental Figure 19A). In contrast, adenocarcinomas showed major upregulation of MAPK3 and CDC7 (Supplemental Figure 19B). These differences likely suggested pathology-specific regulatory mechanisms in kinase signaling networks.

The distribution of ecDNA across histopathologic type

Based on WGS data and the AmpliconArchitect algorithm, the prevalence of ecDNA was comparable between squamous cell carcinoma (38.9%, 44/113) and adenocarcinoma (30.8%, 4/13) (Supplemental Figure 19C). In squamous cell carcinoma samples with ecDNA, hybrid ecDNA alone was the most common type (29 samples, 25.7%), followed by samples containing both hybrid and chromosome ecDNA (10 samples, 8.8%), and chromosome ecDNA alone (5 samples, 4.4%) (Supplemental Figure 19D). Similarly, in adenocarcinoma samples with ecDNA, hybrid ecDNA alone accounted for 2 samples (15.4%), while both hybrid and chromosome ecDNA together represented 1 sample (7.7%), and chromosome ecDNA alone represented 1 sample (7.7%) (Supplemental Figure 19E).

HPV integration patterns across histopathologic type

Among the 662 genomic HPV integration events, 604 integration events were detected in squamous cell carcinomas (Supplemental Figure 19F left) and 46 integration events were detected in adenocarcinomas (Supplemental Figure 19F right). For squamous cell carcinomas, the majority of integration events originated in the HPV E1 region (228/604, 37.7%), followed by L2 (118/604, 19.5%), while the integration events mainly originated in L1 (12/46, 26.1%) and L2 (10/46, 21.7%) for adenocarcinomas. For the human genome, squamous cell carcinomas exhibited a broad distribution of breakpoints, with the higher frequencies observed on chromosomes 14 (59/604, 9.8%), 1 (54/604, 8.9%), and 3 (53/604, 8.8%). Adenocarcinoma breakpoints showed a more

concentrated pattern, predominantly on chromosome 4 (24/46, 52.2%), followed by chromosomes 8 (5/46, 10.9%) and 2 (4/46, 8.7%). In parallel, RNA-sequencing (RNA-seq) identified 126 human-viral fusion transcripts in squamous cell carcinomas (Supplemental Figure 19G left) and 12 human-viral fusion transcripts in adenocarcinomas (Supplemental Figure 19G right). Mapping breakpoint positions along the viral genome revealed that breakpoints were enriched in early regions—most prominently E1 (squamous cell carcinomas, 79/126, 62.7%; adenocarcinomas, 9/12, 75%). For human genome, the breakpoints in squamous cell carcinomas were mainly located on chromosomes 20 (16/126, 12.7%), 3 (14/126, 11.1%) and 6 (13/126, 10.3%), while those of adenocarcinomas were mainly in chromosomes 17 (6/12, 50%) and 4 (4/12, 33.3%). The detailed HPV integration sites were displayed in the Circos plots of Supplemental Figure 19, F and G.

References

1. Alexandrov LB, et al. Signatures of mutational processes in human cancer. *Nature.* 2013;500(7463):415-21.

2. Cancer Genome Atlas Research N, et al. Integrated genomic and molecular characterization of cervical cancer. *Nature.* 2017;543(7645):378-84.

3. Nelson BE, et al. Leveraging TROP2 Antibody-Drug Conjugates in Solid Tumors. *Annu Rev Med.* 2024;75:31-48.

4. Dequeker BJH, et al. MCM complexes are barriers that restrict cohesin-mediated loop extrusion. *Nature.* 2022;606(7912):197-203.

5. Zhou X, et al. MCM2 promotes the stemness and sorafenib resistance of hepatocellular carcinoma cells via hippo signaling. *Cell Death Discov.* 2022;8(1):418.

6. Wang Y, et al. MCM6 is a critical transcriptional target of YAP to promote gastric tumorigenesis and serves as a therapeutic target. *Theranostics.* 2022;12(15):6509-26.

7. Qu K, et al. MCM7 promotes cancer progression through cyclin D1-dependent signaling and serves as a prognostic marker for patients with hepatocellular carcinoma. *Cell Death Dis.* 2017;8(2):e2603.

8. Dongre A, et al. New insights into the mechanisms of epithelial-mesenchymal transition and implications for cancer. *Nat Rev Mol Cell Biol.* 2019;20(2):69-84.

9. Liis Uusküla-Reimand, et al. Untangling the roles of TOP2A and TOP2B in transcription and cancer. *Science advance.* 2022;8(44):eadd4920.

10. Gu L, et al. The Anticancer Activity of a First-in-class Small-molecule Targeting PCNA. *Clin Cancer Res.* 2018;24(23):6053-65.

11. Thorsson V, et al. The Immune Landscape of Cancer. *Immunity.* 2018;48(4):812-30 e14.

Supplemental Figures

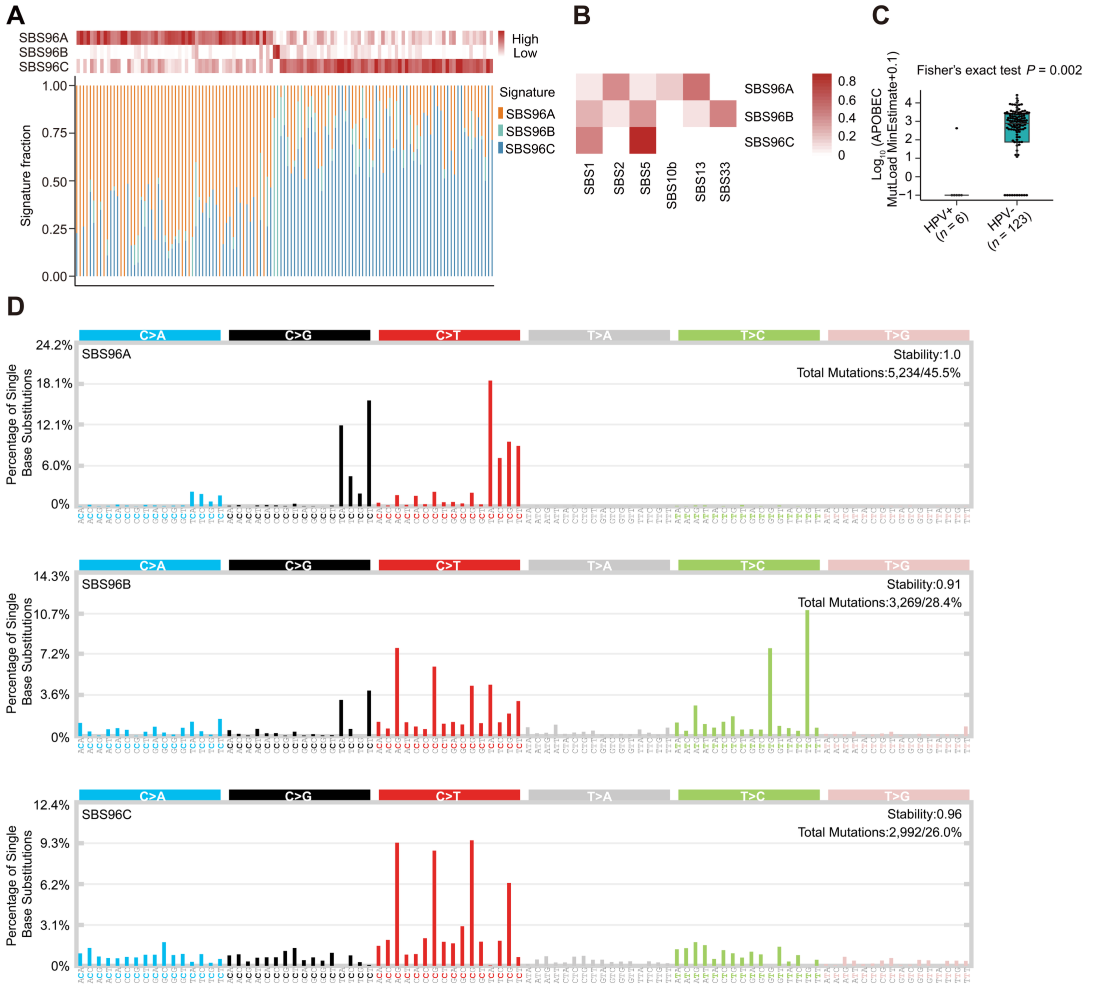

Supplemental Figure 1. Mutation signatures in CC. (**A**) Clustering of CC samples based on relative proportions of SigProfiler-derived mutation signatures. (**B**) Heatmap illustrating correlations between SigProfiler-derived mutation signatures and COSMIC reference signatures. (**C**) Box plot showing the number of APOBEC-induced mutations in HPV+ and HPV- tumors. Centers indicate the medians, the upper and lower boundaries of the boxes indicate the 75th and 25th percentile, whiskers extend to 1.5× interquartile range (IQR), Fisher's exact test, *P* = 0.002. (**D**) Trinucleotide motif frequency plots and enriched mutational signatures identified in CC.

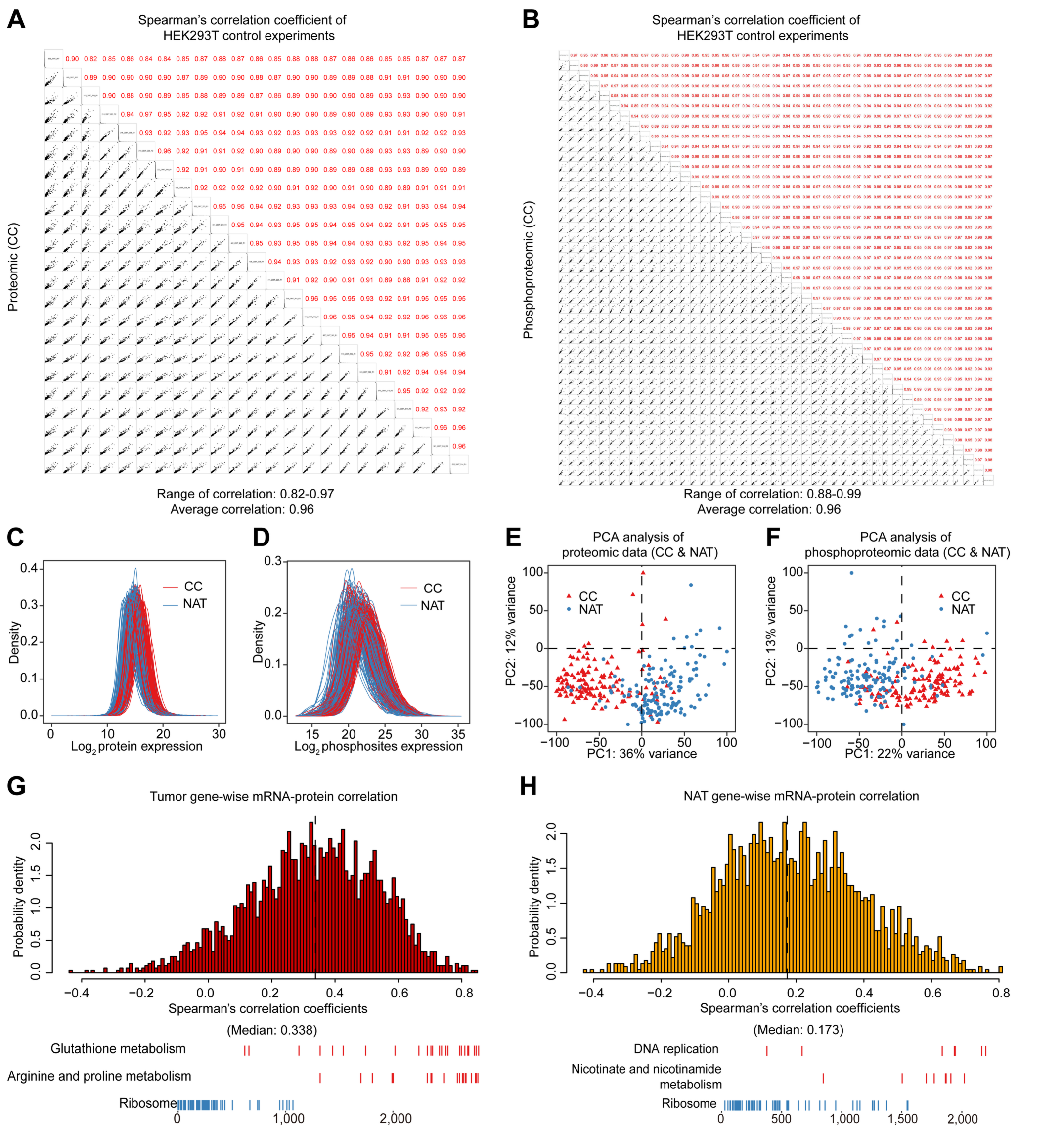

Supplemental Figure 2. Comprehensive quality control and profiling of proteome and phosphoproteome in CC and NAT samples. (**A** and **B**), Quality control of the MS platforms using tryptic digest of HEK293T cells. The top-right half of the panel represents the pairwise Spearman's correlation coefficients of the samples, the bottom-left half of the panel represents the pairwise scatterplots from the same comparison. Proteome **(A)** and Phosphoproteome **(B)** of CC and NAT samples were profiled in Beijing Proteome Research Center, Beijing Institute of Lifeomics, Beijing, China. **(C**

and **D)**, Distribution of protein and phosphoprotein abundances in CC (red) and NAT (blue) samples by density plot. **(E** and **F)**, Principal component analysis of proteomic and phosphoproteomic data in 139 paired CC and NAT samples. **(G** and **H)**, Histograms of gene-wise correlation between mRNA and protein abundances in CC **(G)** and NATs **(H)** (top). Red: pathways in which positively correlated genes were involved; blue: pathways in which negatively correlated genes were involved (bottom).

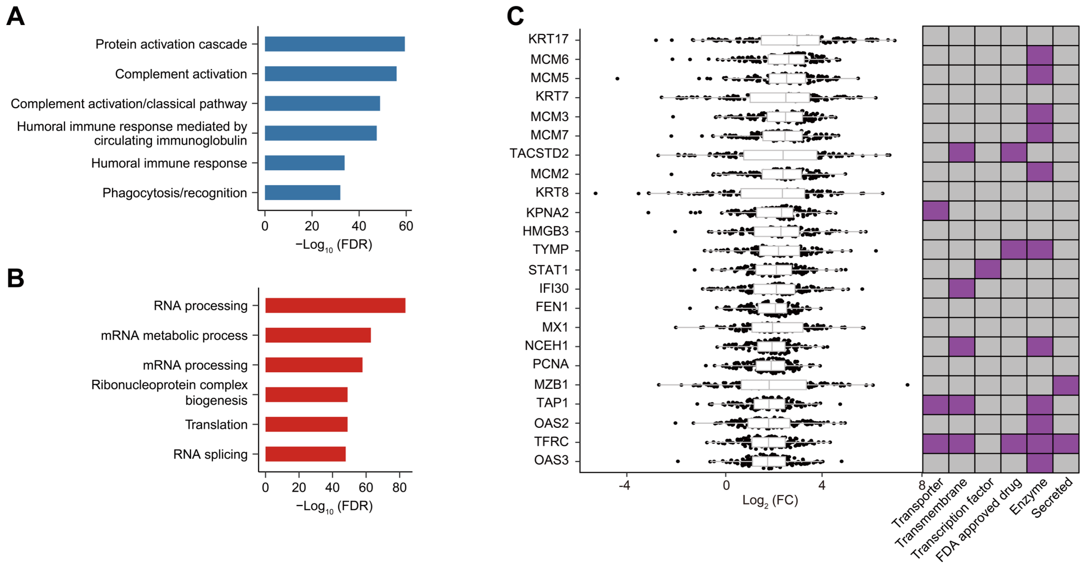

Supplemental Figure 3. (A) GO-BP terms enriched among downregulated proteins in tumors. **(B)** GO-BP terms enriched among upregulated proteins in tumors. **(C)** Log_2-fold-change of proteins in tumors versus paired NATs (left) and corresponding clinical annotations from the Human Protein Atlas (right).

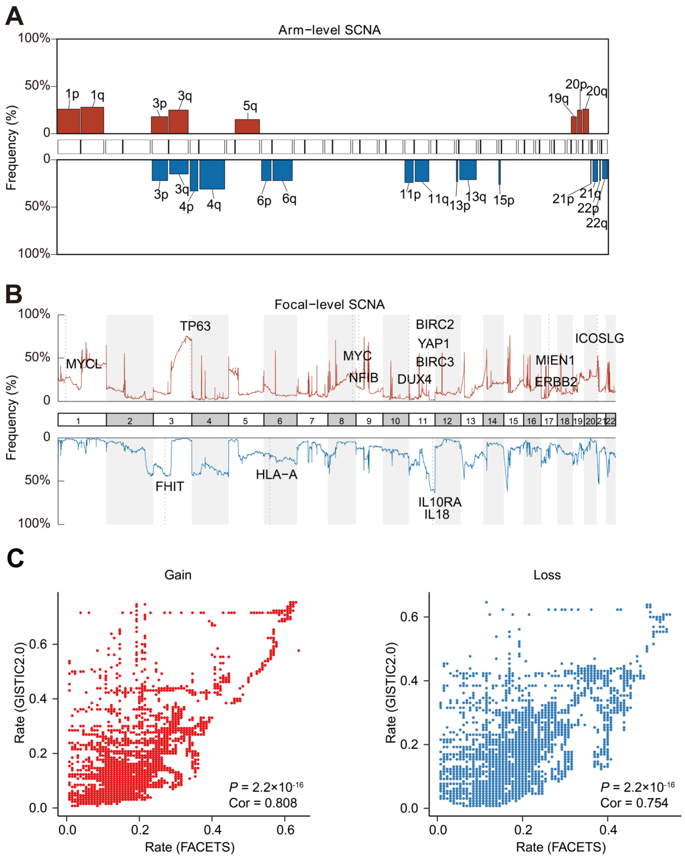

Supplemental Figure 4. SCNAs in CC. (A) Frequency of significant chromosome arm-level SCNA events (Q < 0.25). Red denotes gain and blue denotes loss. **(B)** Frequency of focal-level SCNA events. Focal peaks with significant gains (red) and losses (blue) (Q < 0.25) are shown. Representative novel and reported focal regions and genes encoded from these regions are labeled. (**C**) Concordance between SCNA frequencies inferred by FACETS and GISTIC2.0 for gains (left) and losses (right), Pearson's correlation.

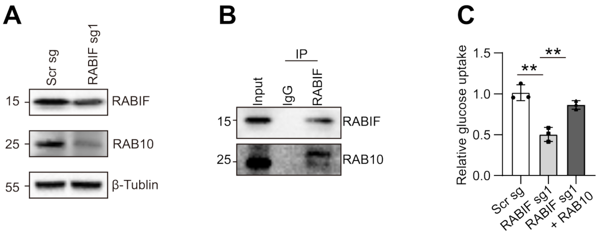

Supplemental Figure 5. RABIF regulated glucose uptake via RAB10. (A) Western blot analysis of RAB10 expression in RABIF knockout SiHa cells. β-Tubulin served as the loading control. **(B)** Immunoprecipitation assay assessing the interaction between RABIF and RAB10 in SiHa cells. **(C)** Glucose uptake measured in SiHa cells. Data are presented as means ± SEM (n = 3 replicates, two-sided Student's t test), *P < 0.05, **P < 0.01, ***P < 0.001.

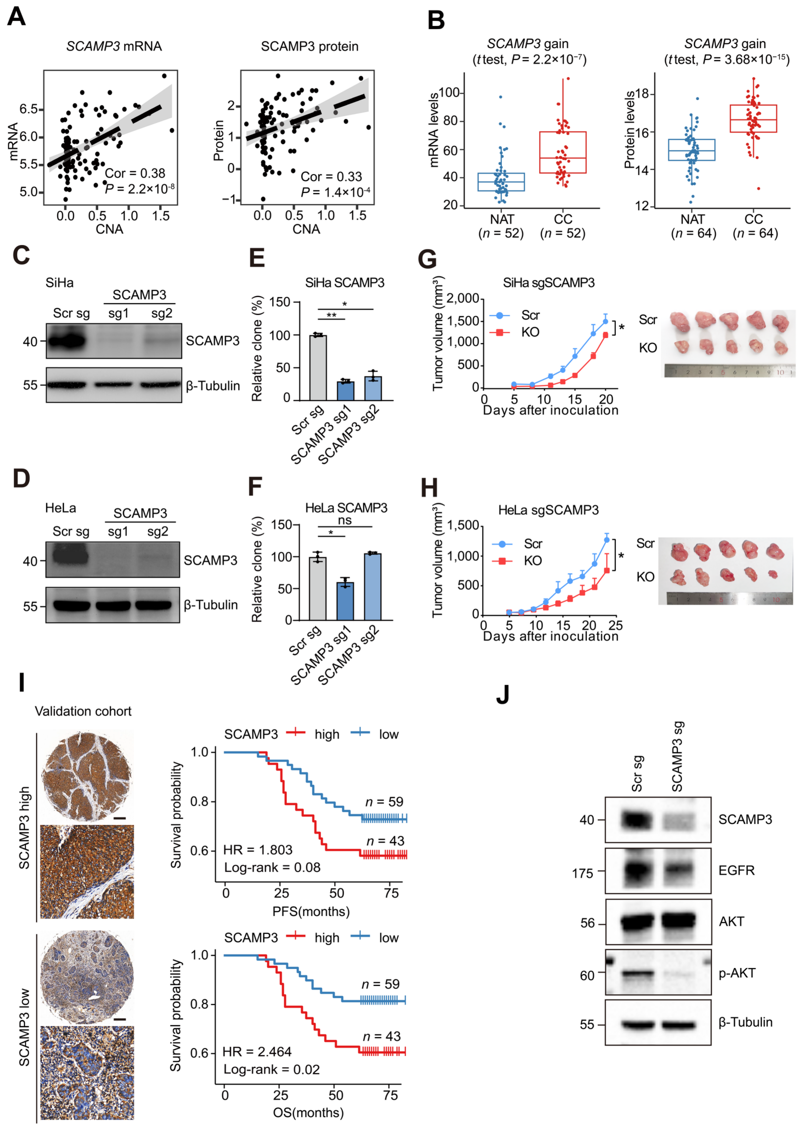

Supplemental Figure 6. Impacts of *SCAMP3* gain on protein expression and tumor growth. (A) Spearman's correlation of *SCAMP3* CNA with mRNA (left) and protein (right) abundances. **(B)** Box-and-whisker plot showing mRNA and protein expression of *SCAMP3* in *SCAMP3* gain CCs and NATs. Centers indicate the medians, the upper and lower boundaries of the boxes indicate the 75th and 25th percentile, whiskers extend to 1.5× interquartile range (IQR); *n* represents the number of samples. **(C and D)** Western blot analysis of *SCAMP3* knockout efficiency in SiHa **(C)** and HeLa **(D)**

cells. **(E and F)** The quantification of *SCAMP3* knockout on colony formation abilities of SiHa **(E)** and HeLa **(F)** cells. Data represent mean ± SEM (n = 3 replicates, two-sided Student's t test. *P < 0.05, **P < 0.01, ***P < 0.001.). **(G and H)** The impacts of *SCAMP3* knockout on tumor growth of SiHa **(G)** and HeLa **(H)** xenograft models. Data represent mean ± SEM (n = 5 mice per group, two-way analysis of variance), *P < 0.05, **P < 0.01, ***P < 0.001. **(I)** Patients in an external cohort (n = 102) were stratified by IHC staining of SCAMP3, representative images of SCAMP3-high and SCAMP3-low were present on the left. Scale bars, 200 μm. Kaplan-Meier curves for PFS (top-right) and OS (bottom-right) of these patients are shown. Statistical analysis was performed using Log-rank tests. **(J)** Western blot analysis of EGFR expression and AKT phosphorylation following SCAMP3 knockout in SiHa cells.

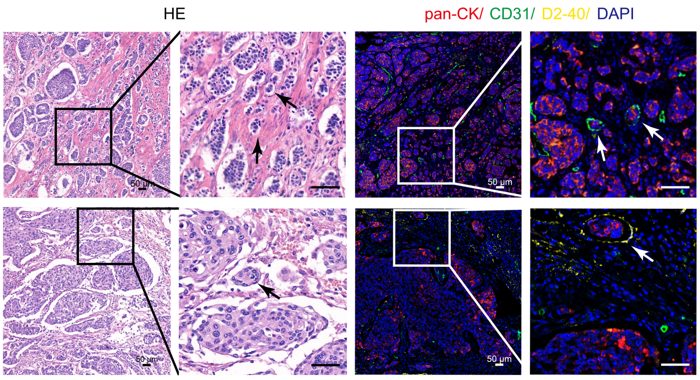

270 **Supplemental Figure 7. Representative multiplexed immunofluorescence images**
271 **showing LVSI in samples of C4.**

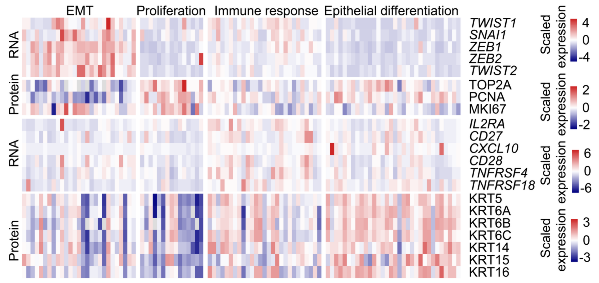

Supplemental Figure 8. Heatmap displays mRNA expression levels of EMT-inducing transcription factors and immune stimulators, along with protein expression levels of proliferation markers and keratins across all 101 tumors.

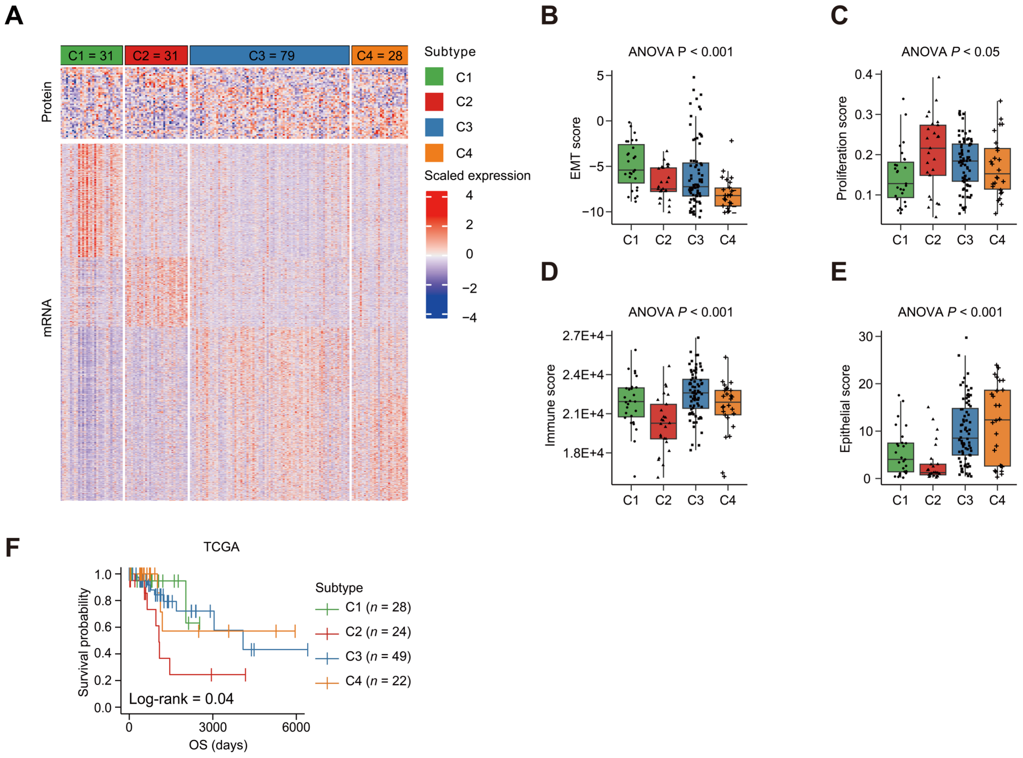

Supplemental Figure 9. Subtyping validation using TCGA dataset. (A) Heatmap of mRNA and protein in TCGA samples across four subtypes. **(B–E)** EMT score **(B)**, proliferation score **(C)**, immune score **(D)**, and epithelial score **(E)** are presented for all TCGA samples (ANOVA test). The middle lines in the boxes are the median, the upper and lower boundaries of the boxes are the first and third quartiles and the whiskers extend to 1.5× the interquartile range of the lower and the upper quartiles. (**F**) Kaplan-Meier curves comparing OS across all TCGA samples (Log-rank tests), *n* represents the number of samples.

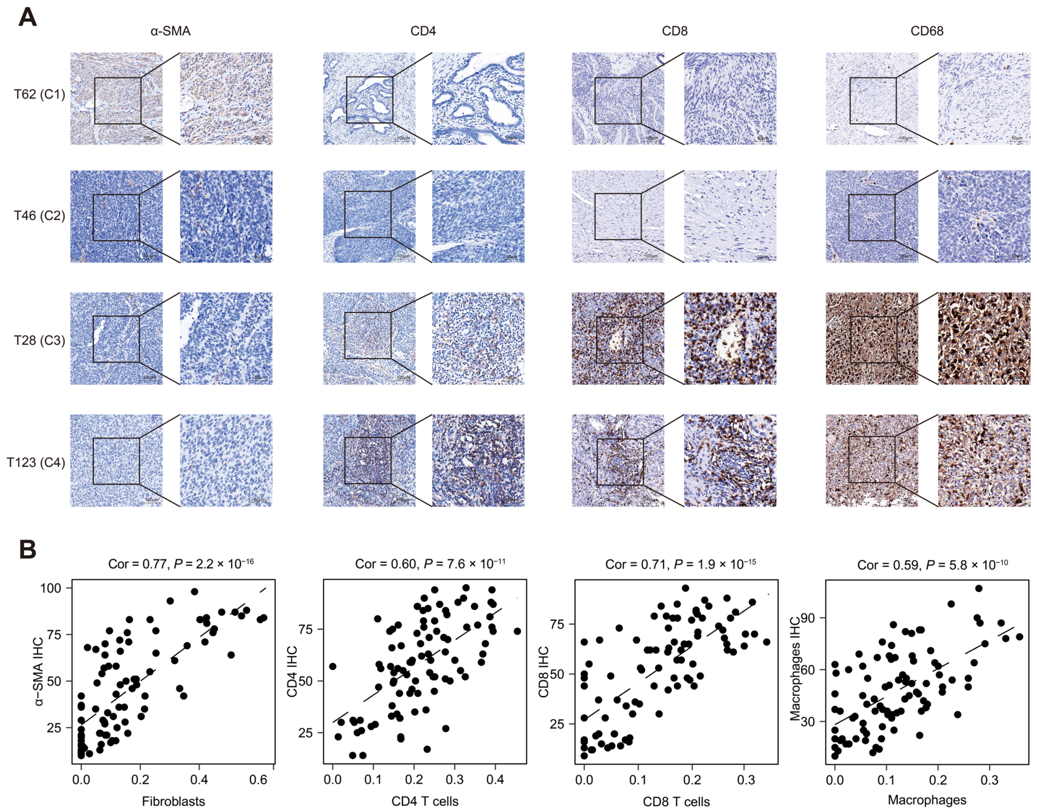

Supplemental Figure 10. Validation of fibroblastic and lymphocytic infiltrates by IHC. (A) IHC staining images of fibroblasts and immune cell markers for 4 tumor samples. **(B)** Correlation between IHC scores of immune-related markers (y axis) and the percentages of the corresponding cell type (x axis) estimated by xCell in 93 tumor samples, Cor > 0.55, P < 0.05. The correlations were assessed using Spearman's rank-order correlation analysis.

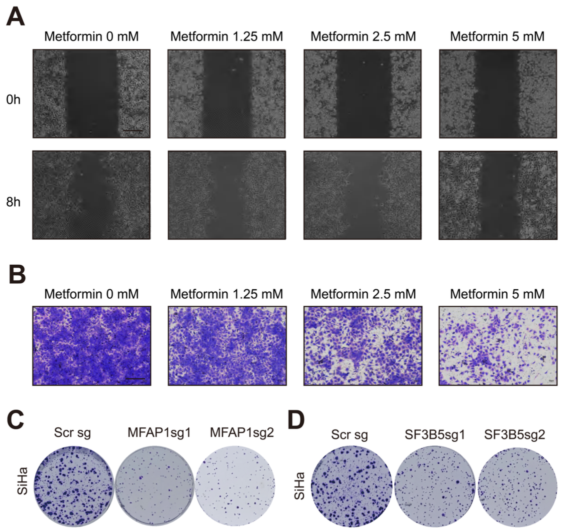

Supplemental Figure 11. (**A**) Representative images of transwell migration assays in S12 cells treated with Metformin (*n* = 3 biological replicates). Scale bar, 200 μm. (**B**) Representative images of wound healing assays in S12 cells, measured at 0 h and 8 h after injury (*n* = 3 biological replicates). Scale bar, 200 μm. (**C** and **D**) The impacts of MFAP1 (**C**) and SF3B5 (**D**) knockout on colony formation in SiHa cells.

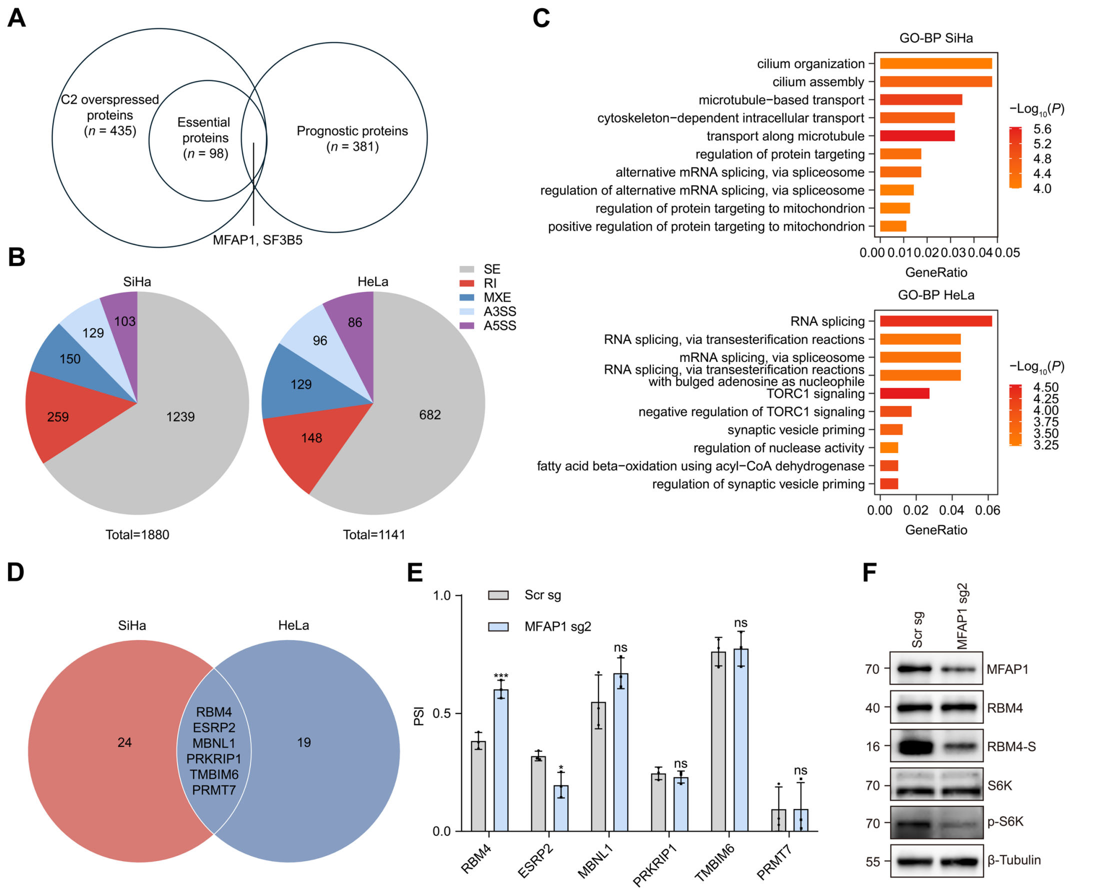

Supplemental Figure 12. Profiles and validations of MFAP1-affected AS events in CC cells. (A) Number of proteins classified as C2 special, essential, or prognostic alone, or in combination, with MFAP1 and SF3B5 shared across all three categories. **(B)** MFAP1-affected alternative splicing (AS) events in SiHa (left) and HeLa (right) cell lines. The AS events are classified into 5 categories: skipped exon (SE), retained intron (RI), mutually-exclusive exon (MXE), alternative 3'splice site (A3SS), alternative 5'splice site (A5SS). **(C)** GO-BP terms enriched among AS genes in SiHa and HeLa cell lines. **(D)** Overlapping AS targets enriched in RNA splicing between SiHa and HeLa cell lines. **(E)** RT-PCR validation of the six overlapping AS targets. ImageJ software was used to quantify the gray intensity of the DNA gel band and PSI was calculated as splice_in / (splice_in + splice_out) (n = 3 replicates, two-sided Student's

t test), **P* < 0.05, ***P* < 0.01, ****P* < 0.001. **(F)** Western blot analysis of MFAP1, RBM4, RBM4-S, S6K and p-S6K in SiHa cells.

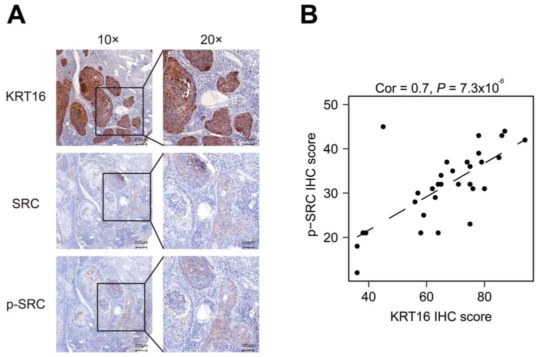

Supplemental Figure 13. Correlation between KRT16 and p-Src in C4 subtype samples. (A) Representative IHC staining of KRT16, Src and p-Src. **(B)** Correlation between IHC scores of p-Src (y axis) and KRT16 (x axis). Cor = 0.64; $P = 7.3 \times 10^{-06}$.

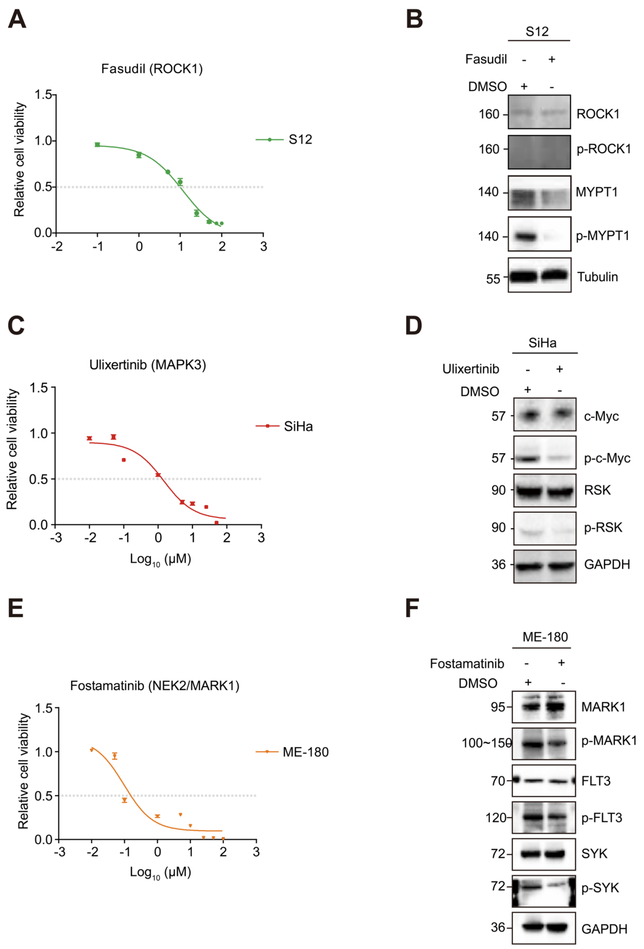

Supplemental Figure 14. Validation of kinase inhibitor effects downstream signaling. (**A**) Relative viability of SiHa cells treated with Ulixertinib for 72h. (**B**) Western blot analysis of the changes in C-MYC and RSK phosphorylation in SiHa cells treated with Ulixertinib for 24h. GAPDH served as a loading control. (**C**) Relative viability of ME-180 cells treated with Fostamatinib for 72h. (**D**) Western blot analysis of the changes in MARK and FLT3 phosphorylation in ME-180 cells treated with Fostamatinib for 24h. GAPDH served as a loading control.

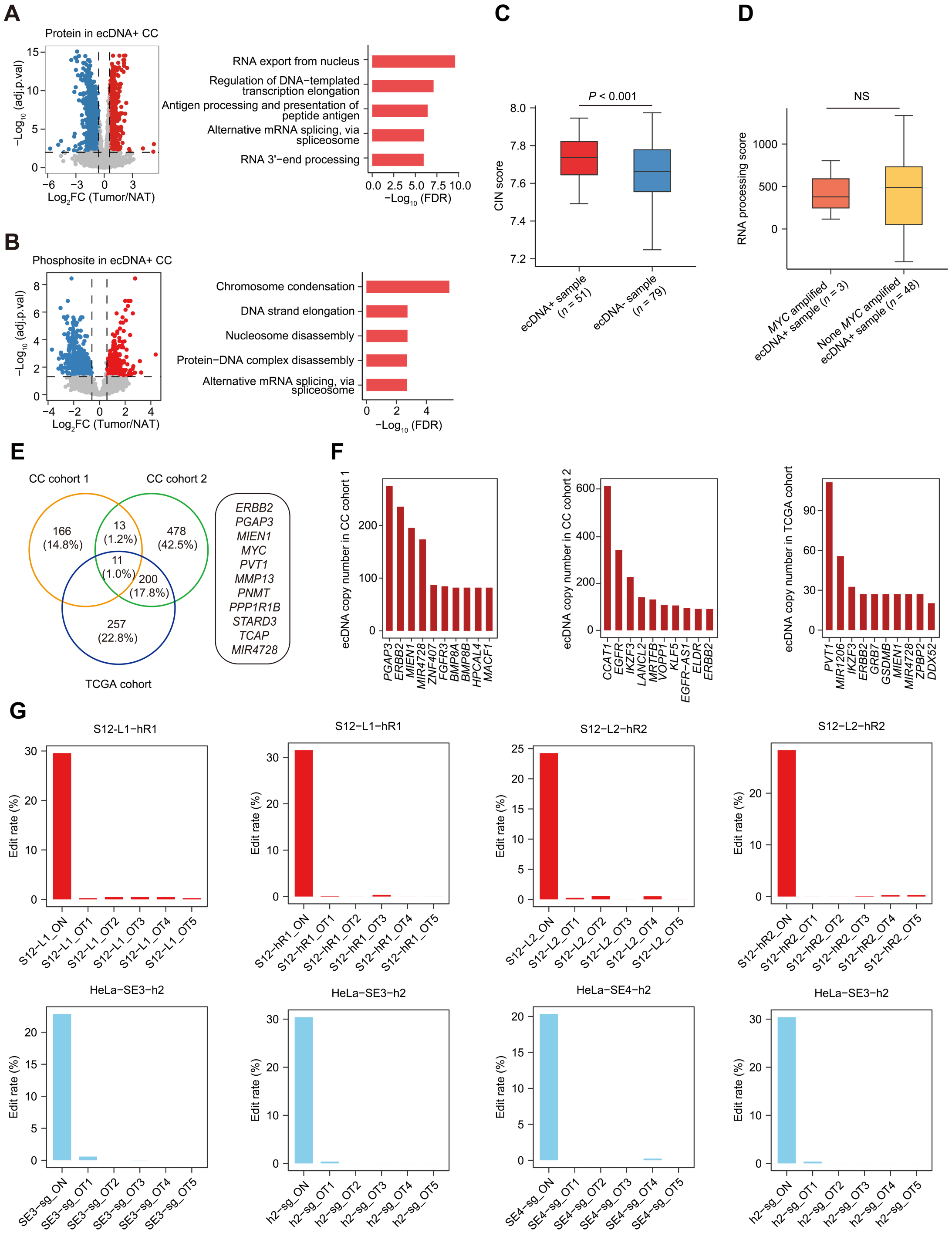

Supplemental Figure 15. ecDNA landscape of CC cohorts. (A) Volcano plot depicting proteins differentially expressed in tumors and NATs of ecDNA+ samples (BH adjusted P value < 0.01, fold-change > 1.5, modified t test) (left). Enriched GO terms for the increased proteins in tumors (right). **(B)** Volcano plot illustrating

differentially expressed phosphosites in tumors and the paired NATs of ecDNA+ samples (BH adjusted P value < 0.05, fold-change > 1.5, modified t test) (left). Enriched GO terms for the corresponding proteins of increased phosphosites in tumors (right). (**C**) Box plot showing the differences in CIN scores between ecDNA+ and ecDNA- samples. (**D**) Box plot showing the differences in RNA processing scores between *MYC* amplified ecDNA+ samples and none *MYC* amplified ecDNA+ samples. (**E**) Venn plot illustrating shared and unique ecDNA-associated genes across the three cohorts. (**F**) Gene-wise ecDNA copy number distributions in CC cohort 1, CC cohort 2 and the TCGA cohort. (**G**) On-target and off-target editing rates in HeLa and S12 cell lines.

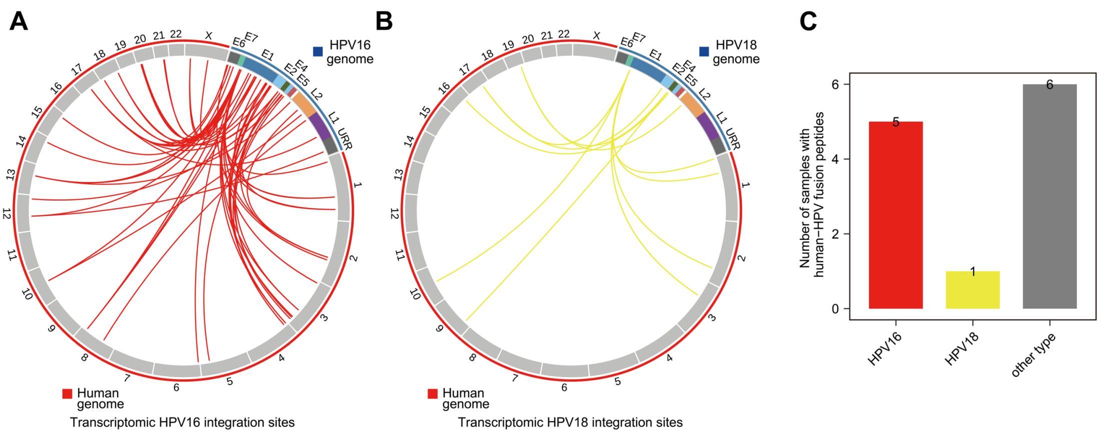

Supplemental Figure 16. Human–HPV fusion transcripts and fusion peptides, stratified by HPV type. (**A**–**B**) Joint Circos plot showing human-HPV fusion transcript breakpoints from the HPV16 genome (**A**) and HPV18 genome (**B**) to the human genome. (**C**) Distribution of HPV type in human-HPV fusion peptides.

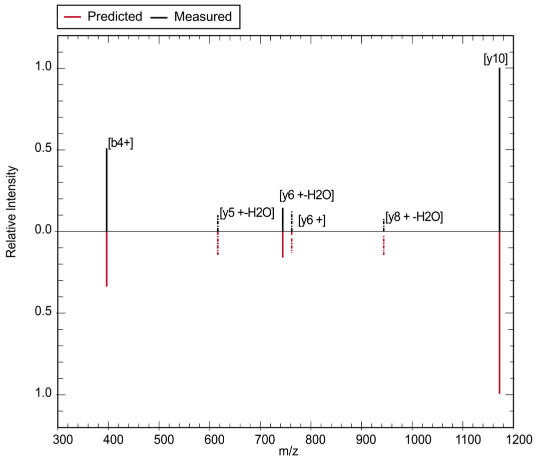

Supplemental Figure 17. The predicted spectrum and measured spectrum of[Acetyl(ProteinNterm)]ADPAASGSYPRNSGVLQNPSLQTTRTR.3 in sample T115.

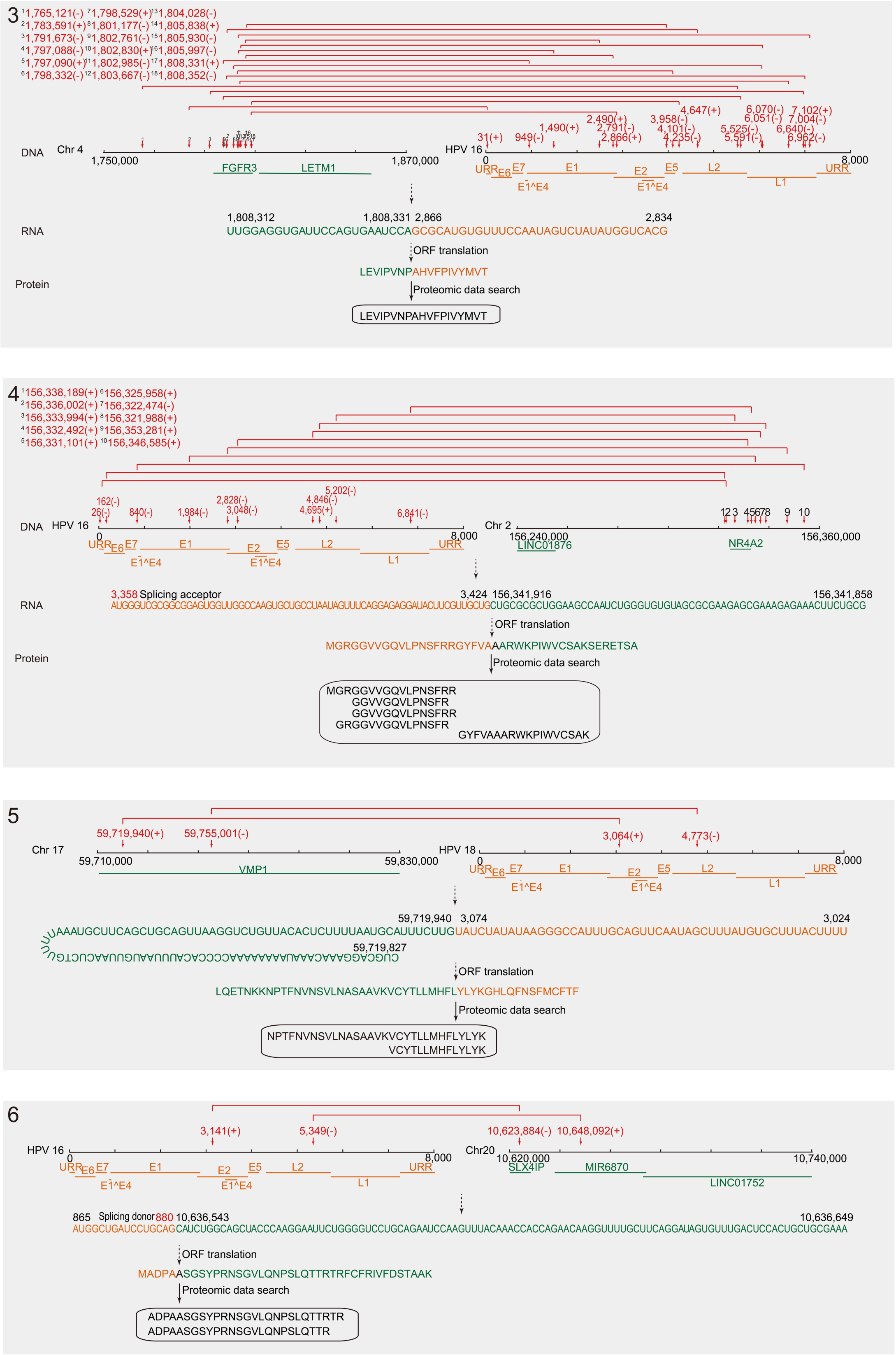

Supplemental Figure 18. Detailed information for the human-viral fusion peptides.

Genomic breakpoints, ORF of elongated RNA fusion reads, predicted fusion peptides

and detected fusion peptides of peptides No. 3–6

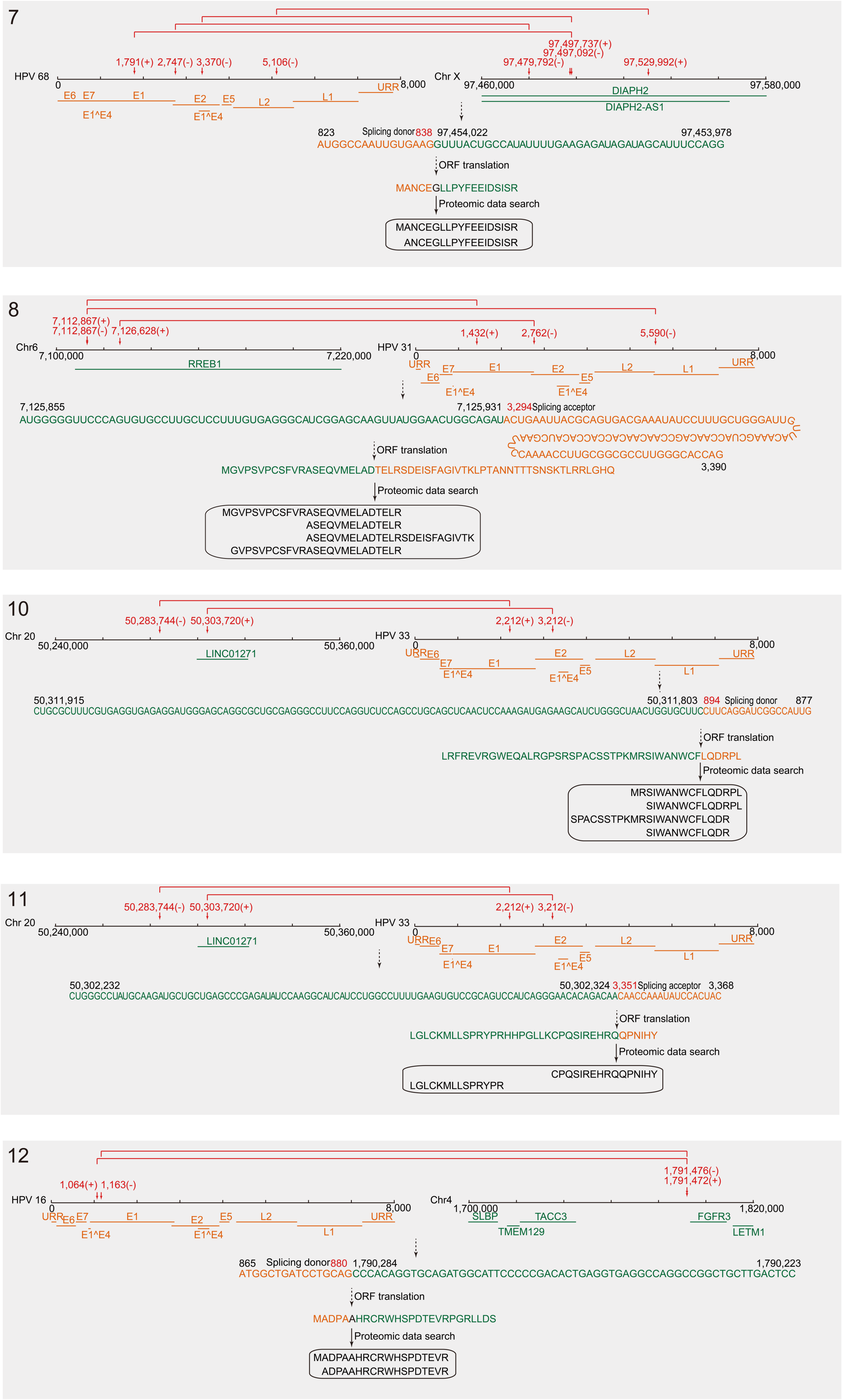

7
HPV 68
1,791(+) 2,747(-) 3,370(-) 5,106(-)
0
8,000
E6 E7 E1 E2 E5 L2 L1 URR
E1^E4 E1^E4
Chr X
97,497,737(+)
97,497,092(-)
97,479,792(-)
97,529,992(+)
97,460,000
97,580,000
DIAPH2
DIAPH2-AS1
823 Splicing donor 838 97,454,022 97,453,978
AUGGCCAAUUGUGAAGGUUUACUGCCAUAUUUUGAAGAGAUAGAUAGCAUUUCCAGG
ORF translation
MANCEGLLPYFEEIDSISR
Proteomic data search
MANCEGLLPYFEEIDSISR
ANCEGLLPYFEEIDSISR
8
7,112,867(+)
7,112,867(-) 7,126,628(+)
Chr6
7,100,000
7,220,000
RREB1
HPV 31
1,432(+) 2,762(-) 5,590(-)
0
8,000
URR E6 E7 E1 E2 E5 L2 L1 URR
E1^E4 E1^E4
7,125,855
7,125,931 3,294 Splicing acceptor
3,390
ORF translation
MGVPSVPCSFVRASEQVMELADTELRSDEISFAGIVTKLPTANNTTTSNSKTLRRLGHQ
Proteomic data search
MGVPSVPCSFVRASEQVMELADTELR
ASEQVMELADTELR
ASEQVMELADTELRSDEISFAGIVTK
GVPSVPCSFVRASEQVMELADTELR
10
Chr 20
50,283,744(-) 50,303,720(+)
50,240,000
50,360,000
LINC01271
HPV 33
2,212(+) 3,212(-)
0
8,000
URR E6 E7 E1 E2 E5 L2 L1 URR
E1^E4 E1^E4
50,311,915
50,311,803 894 Splicing donor 877
ORF translation
LRFREVRGWEQALRGPSRSPACSSTPKMRSIWANWCFLQDRPL
Proteomic data search
MRSIWANWCFLQDRPL
SIWANWCFLQDRPL
SPACSSTPKMRSIWANWCFLQDR
SIWANWCFLQDR
11
Chr 20
50,283,744(-) 50,303,720(+)
50,240,000
50,360,000
LINC01271
HPV 33
2,212(+) 3,212(-)
0
8,000
URR E6 E7 E1 E2 E5 L2 L1 URR
E1^E4 E1^E4
50,302,232
50,302,324 3,351 Splicing acceptor 3,368
ORF translation
LGLCKMLLSPRYPRHHPGLLKCPQSIREHRQQPNIHY
Proteomic data search
CPQSIREHRQQPNIHY
LGLCKMLLSPRYPR
12
HPV 16
1,064(+) 1,163(-)
0
8,000
URR E6 E7 E1 E2 E5 L2 L1 URR
E1^E4 E1^E4
Chr4
1,791,476(-)
1,791,472(+)
1,700,000
1,820,000
SLBP TACC3 FGFR3
TMEM129 LETM1
865 Splicing donor 880 1,790,284 1,790,223
ATGGCTGATCCTGCAGCCCACAGGTGCAGATGGCATTCCCCCGACACTGAGGTGAGGCCAGGCCGGCTGCTTGACTCC
ORF translation
MADPAAHRCRWHSPDTEVRPGRLLDS
Proteomic data search
MADPAAHRCRWHSPDTEVR
ADPAAHRCRWHSPDTEVR

Supplemental Figure 18. Detailed information for the human-viral fusion peptides. Genomic breakpoints, ORF of elongated RNA fusion reads, predicted fusion peptides and detected fusion peptides of peptides No. 7–8, 10–12.

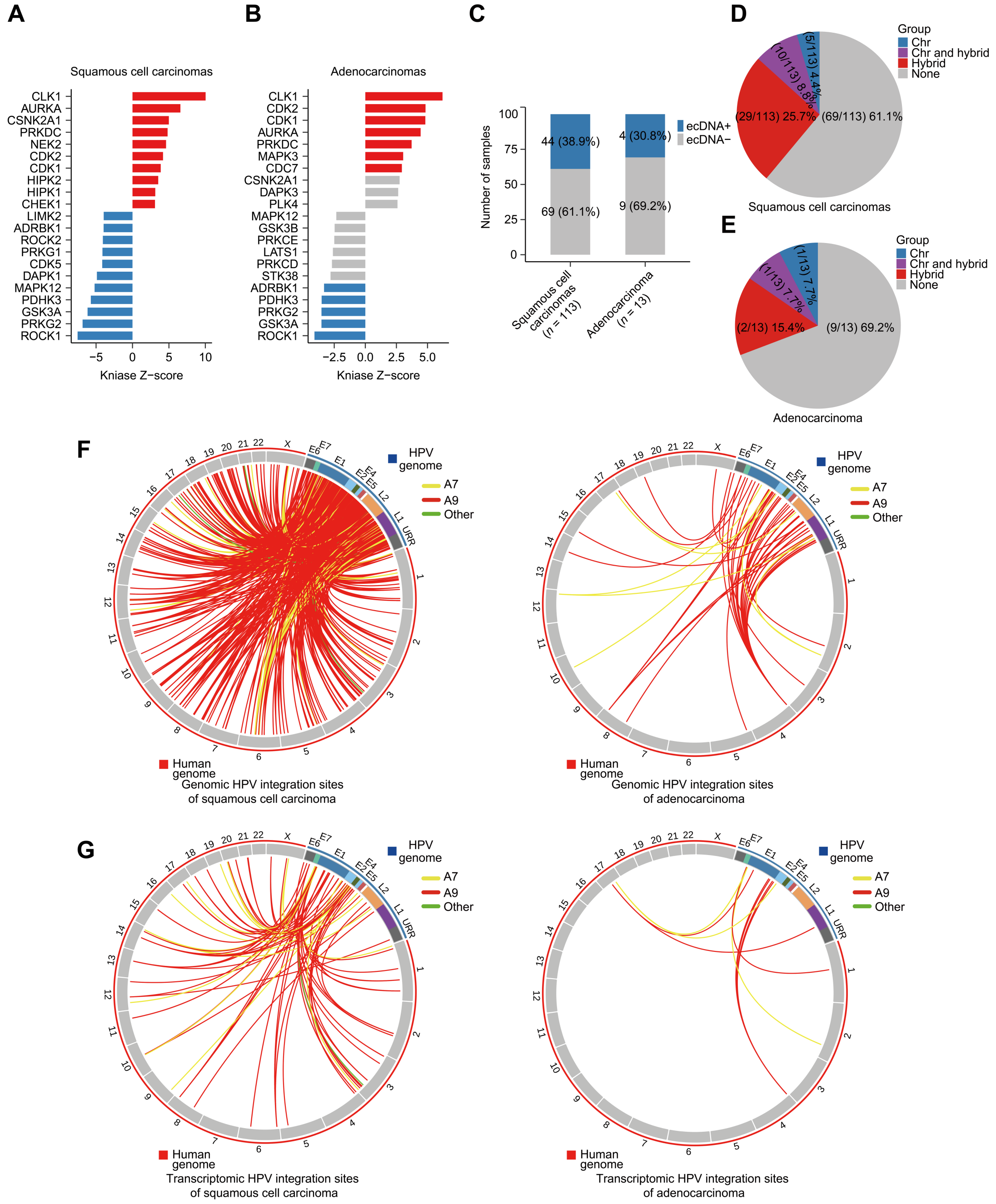

344 **Supplemental Figure 19. Analysis of the main results stratified by major**
345 **histopathologic type squamous cell carcinoma and adenocarcinoma.** (**A–B**)
346 Evaluation of kinase activities by KSEA in squamous cell carcinomas (**A**) and
347 adenocarcinoma (**B**). The distribution of whole ecDNA (**C**), hybrid ecDNA and
348 chromosome ecDNA in squamous cell carcinomas (**D**) and adenocarcinomas (**E**). Joint
349 Circos plot of HPV genes in genomic HPV integration sites (**F**) and transcriptomic HPV

integration sites (**G**).
