# Supplementary material for: Proteogenomic characterization of cervical cancer identifies molecular subtypes predictive of clinical outcomes and subtype-specific targets
Source: J Clin Invest. 2026 Feb 10;136(7):e199497. doi: 10.1172/JCI199497 (PMC13038215; doi:10.1172/JCI199497)

Full unedited gel for Figure 2H

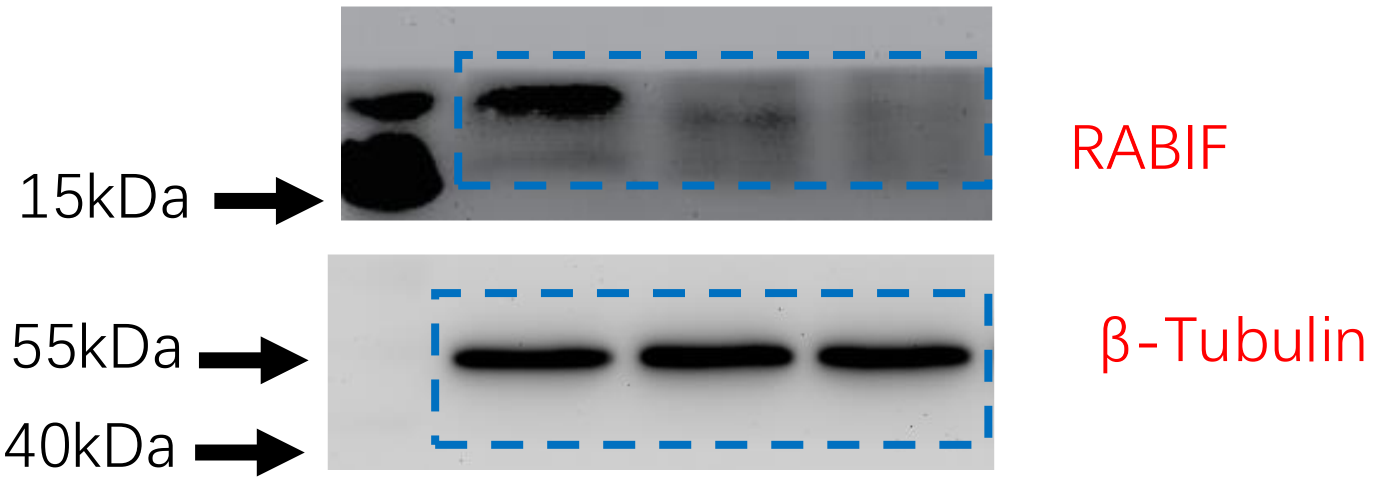

Full unedited gel for Figure 2I

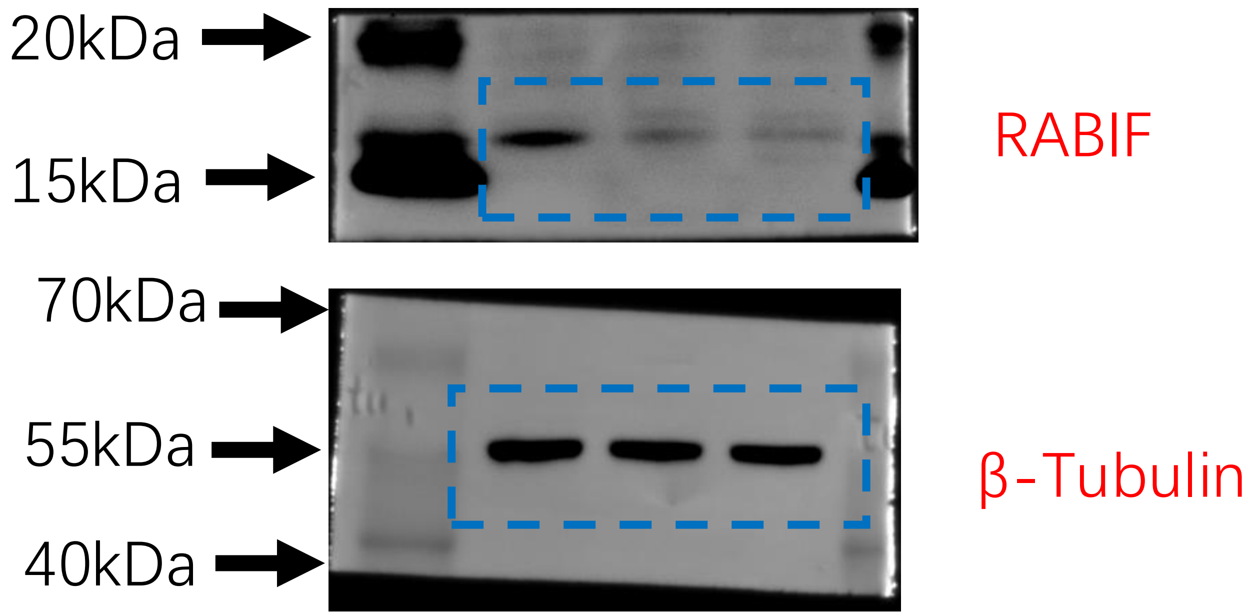

Full unedited gel for Figure 5H

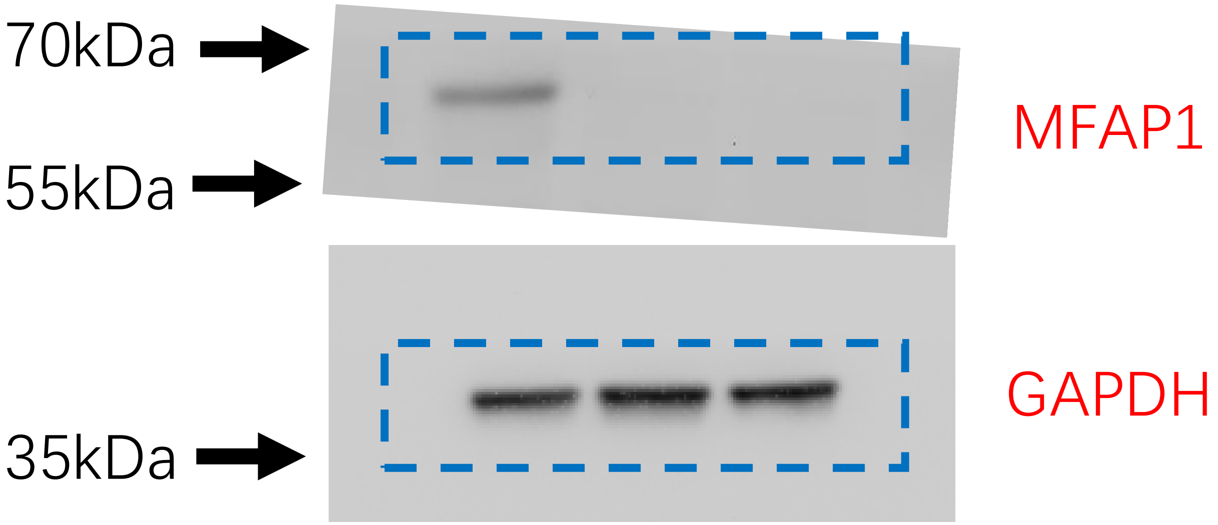

Full unedited gel for Figure 5K

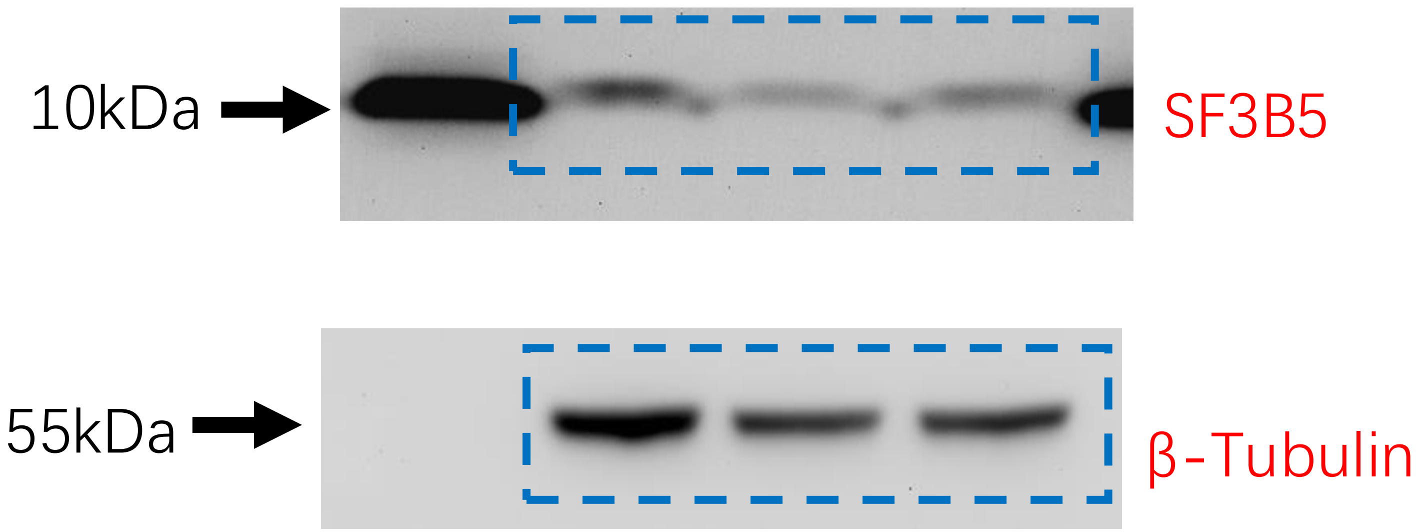

Full unedited gel for Figure 5Q

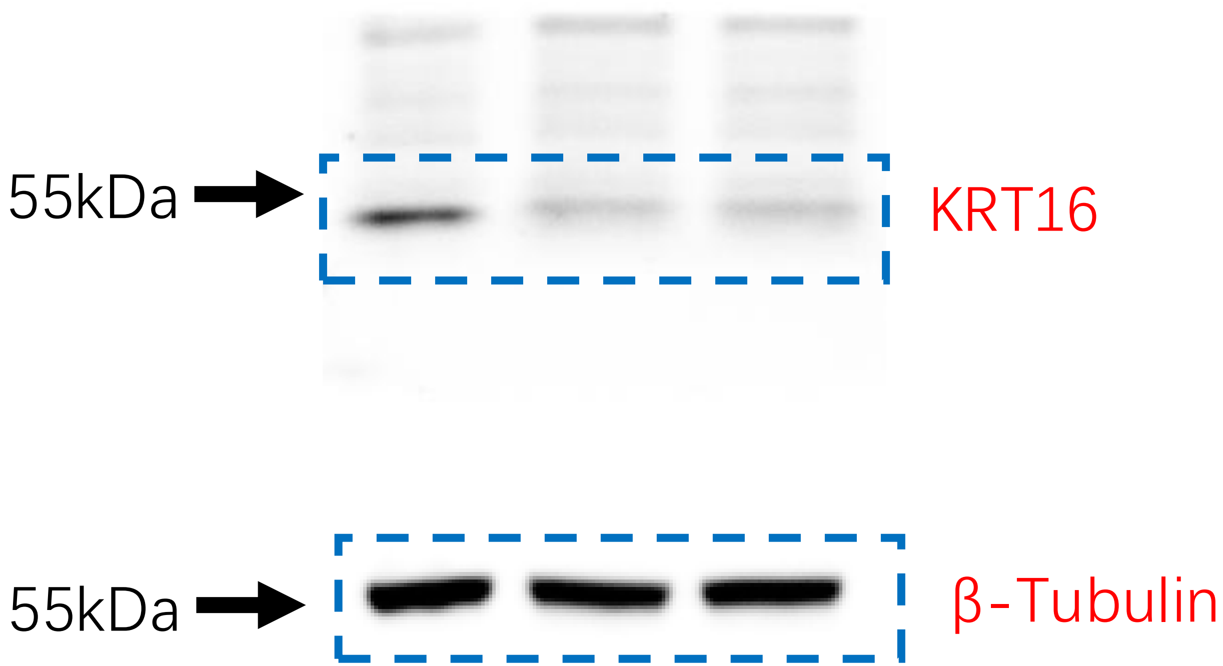

Full unedited gel for Supplemental Figure 5A

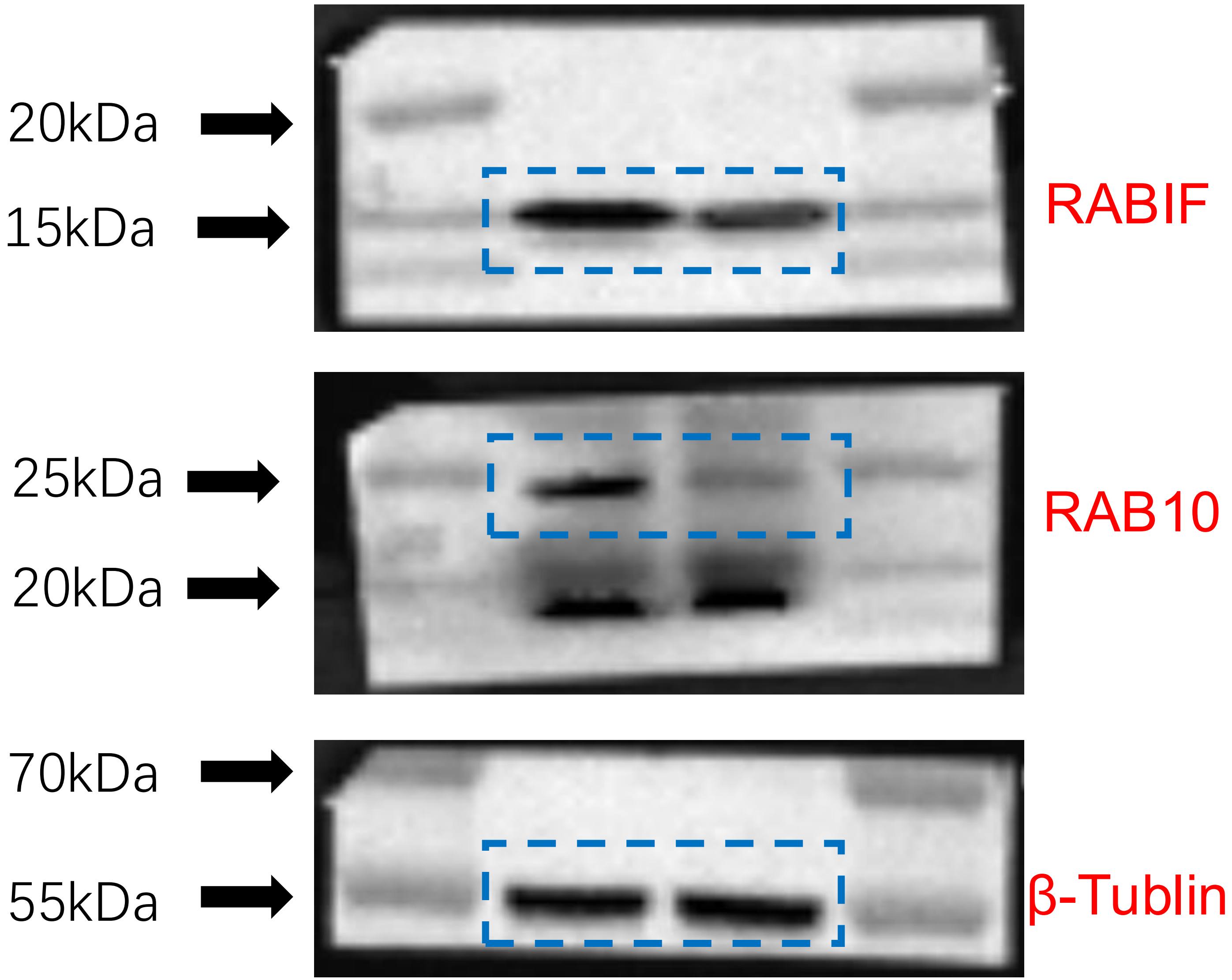

Full unedited gel for Supplemental Figure 5B

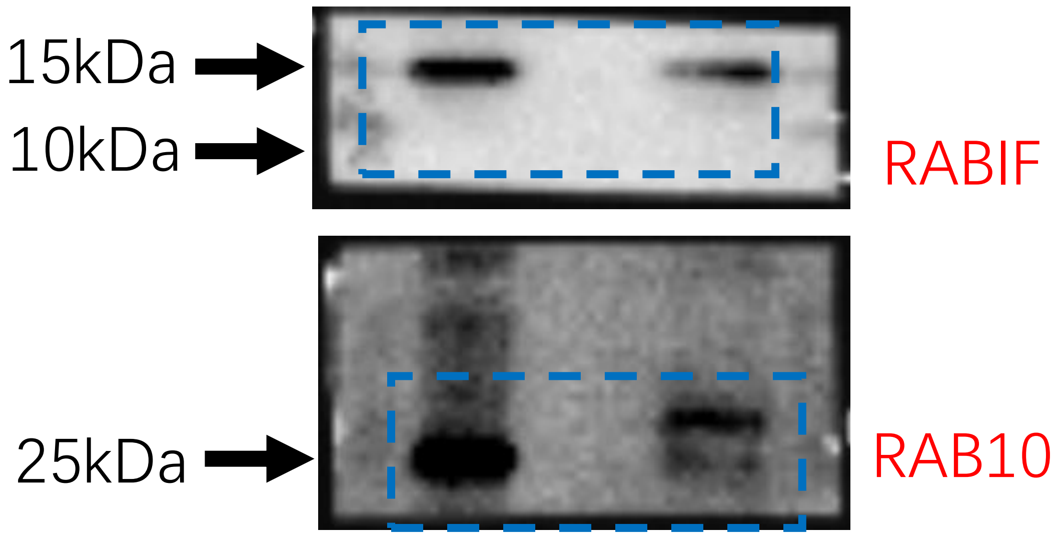

Full unedited gel for Supplemental Figure 6C

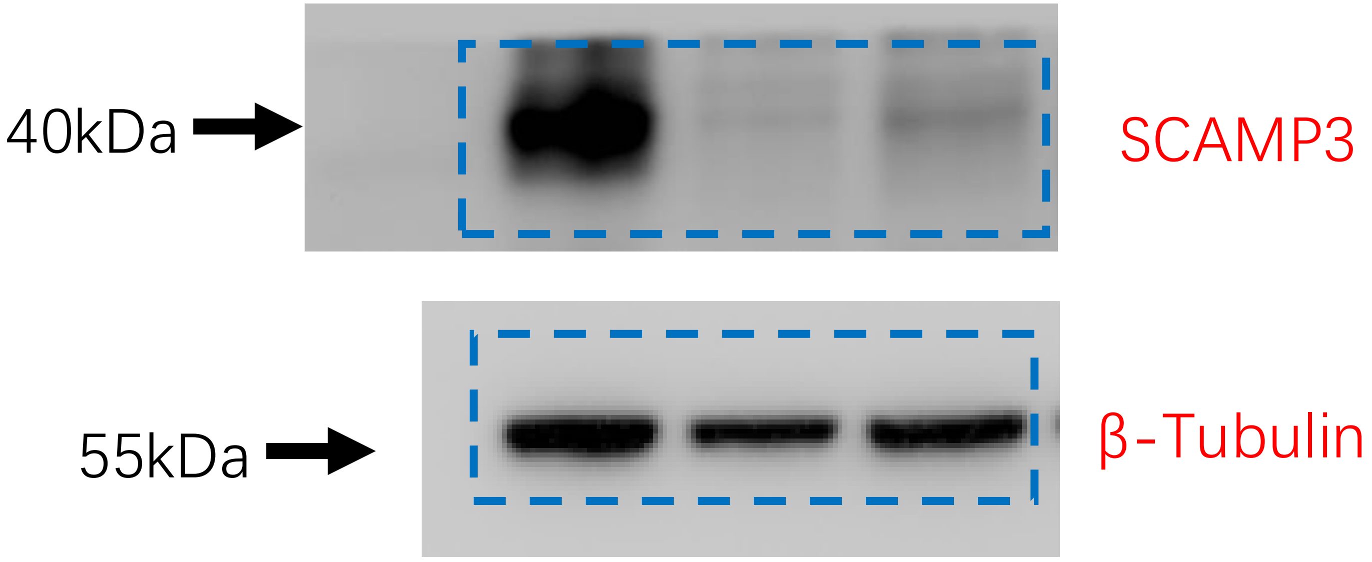

Full unedited gel for Supplemental Figure 6D

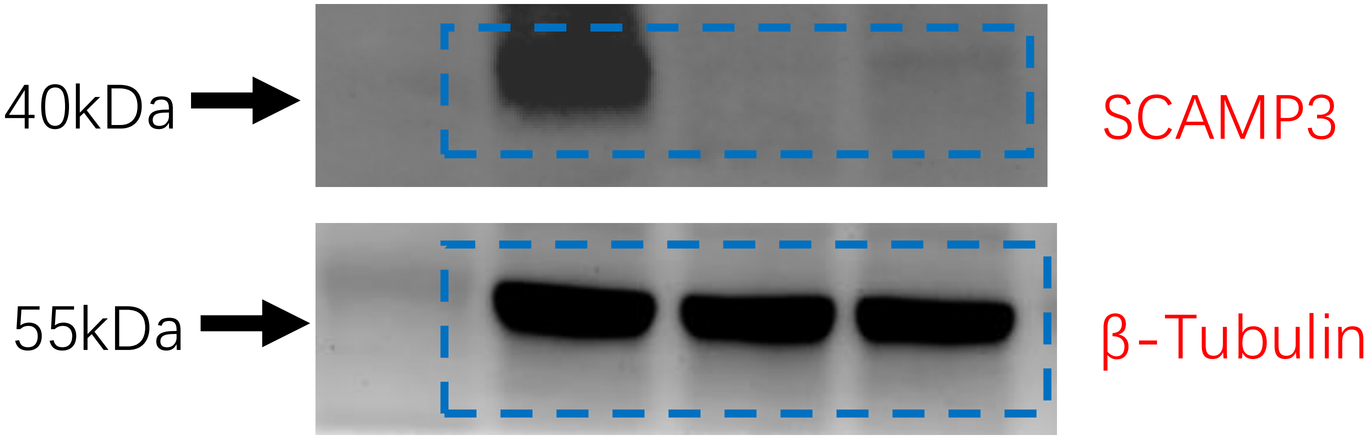

Full unedited gel for Supplemental Figure 6J

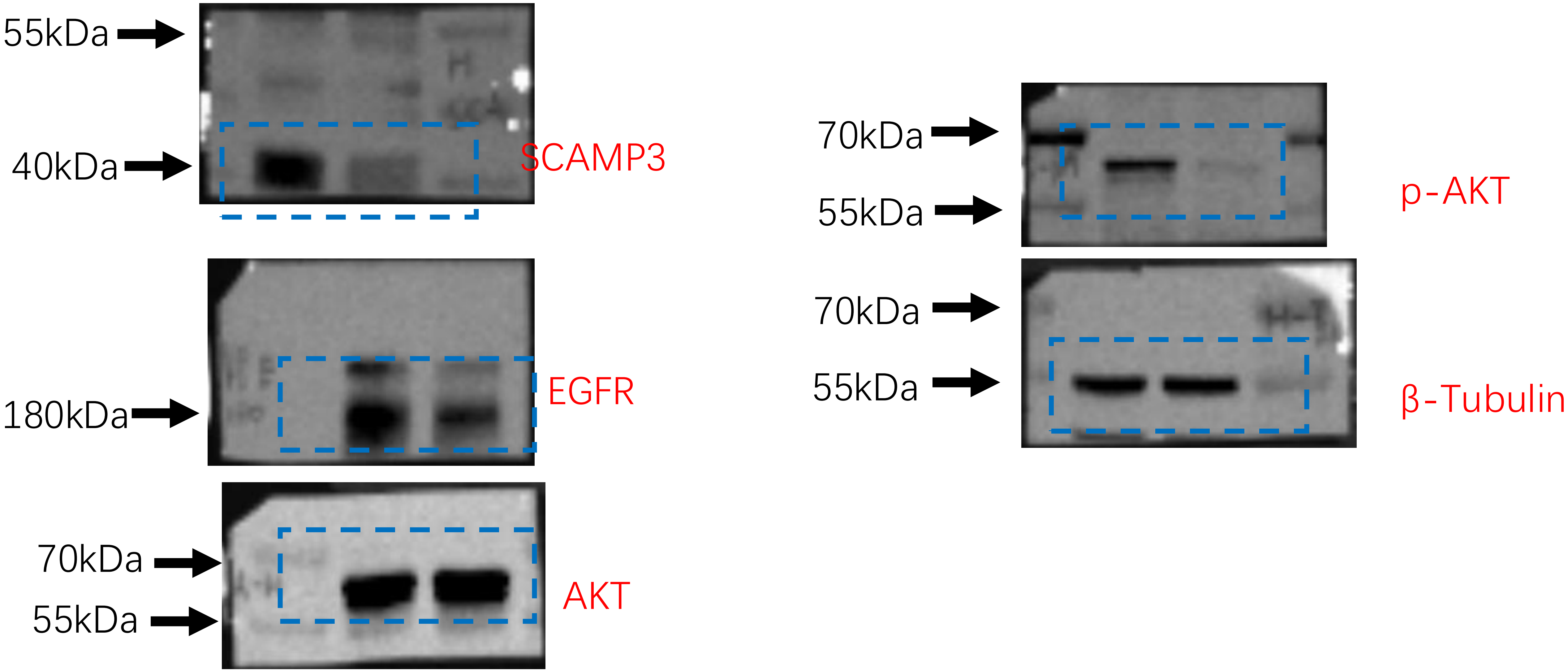

Full unedited gel for Supplemental Figure 12F

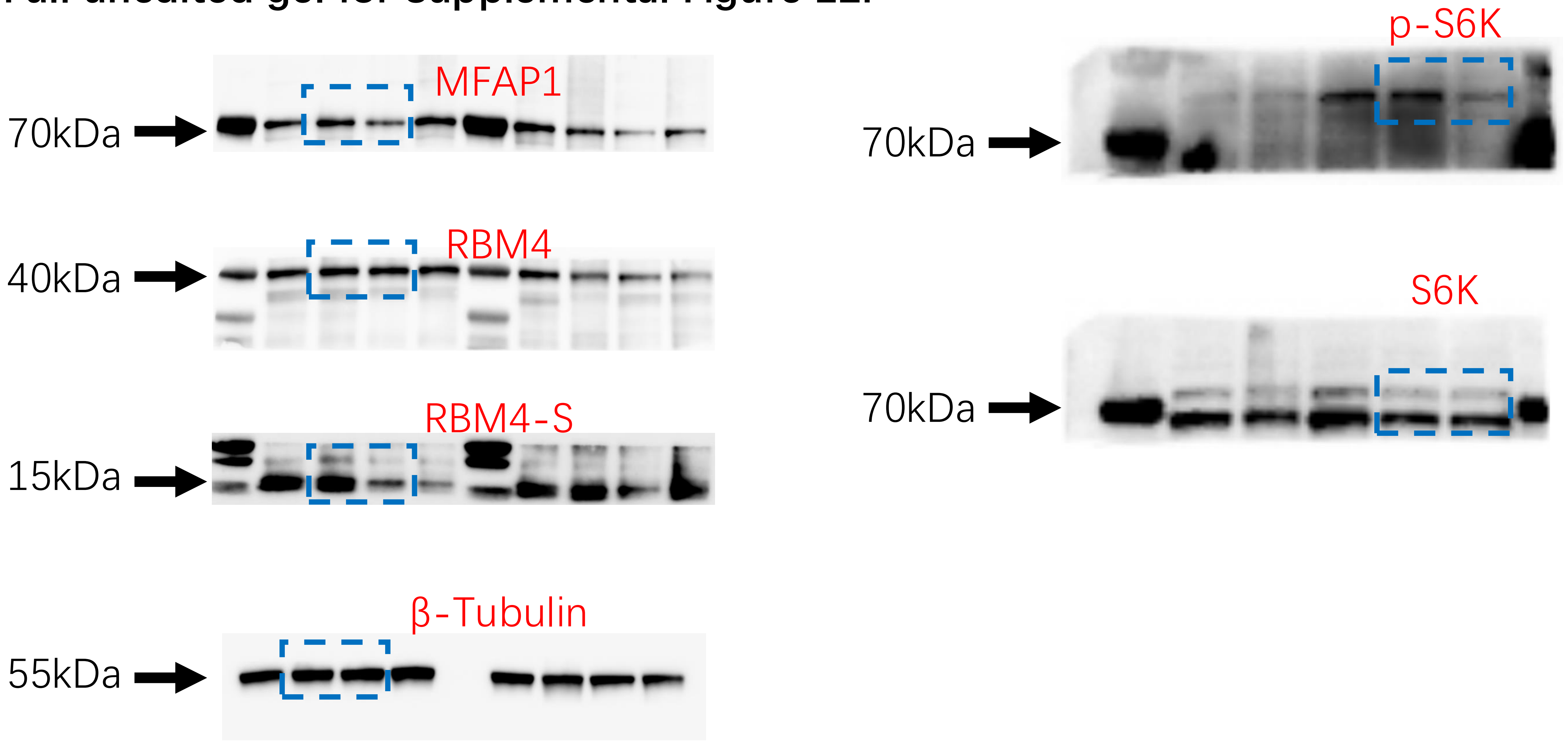

Full unedited gel for Supplemental Figure 14B

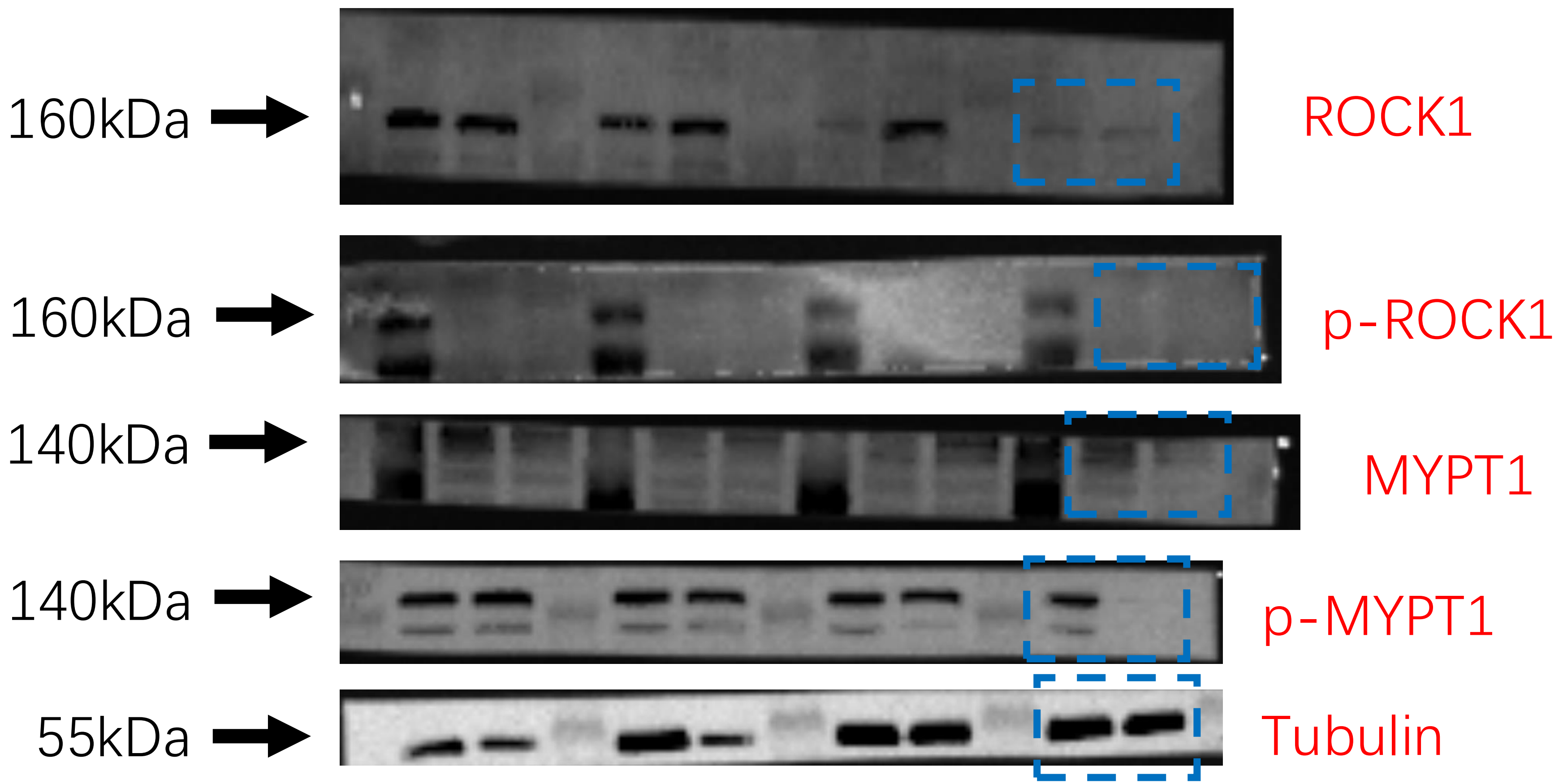

Full unedited gel for Supplemental Figure 14C

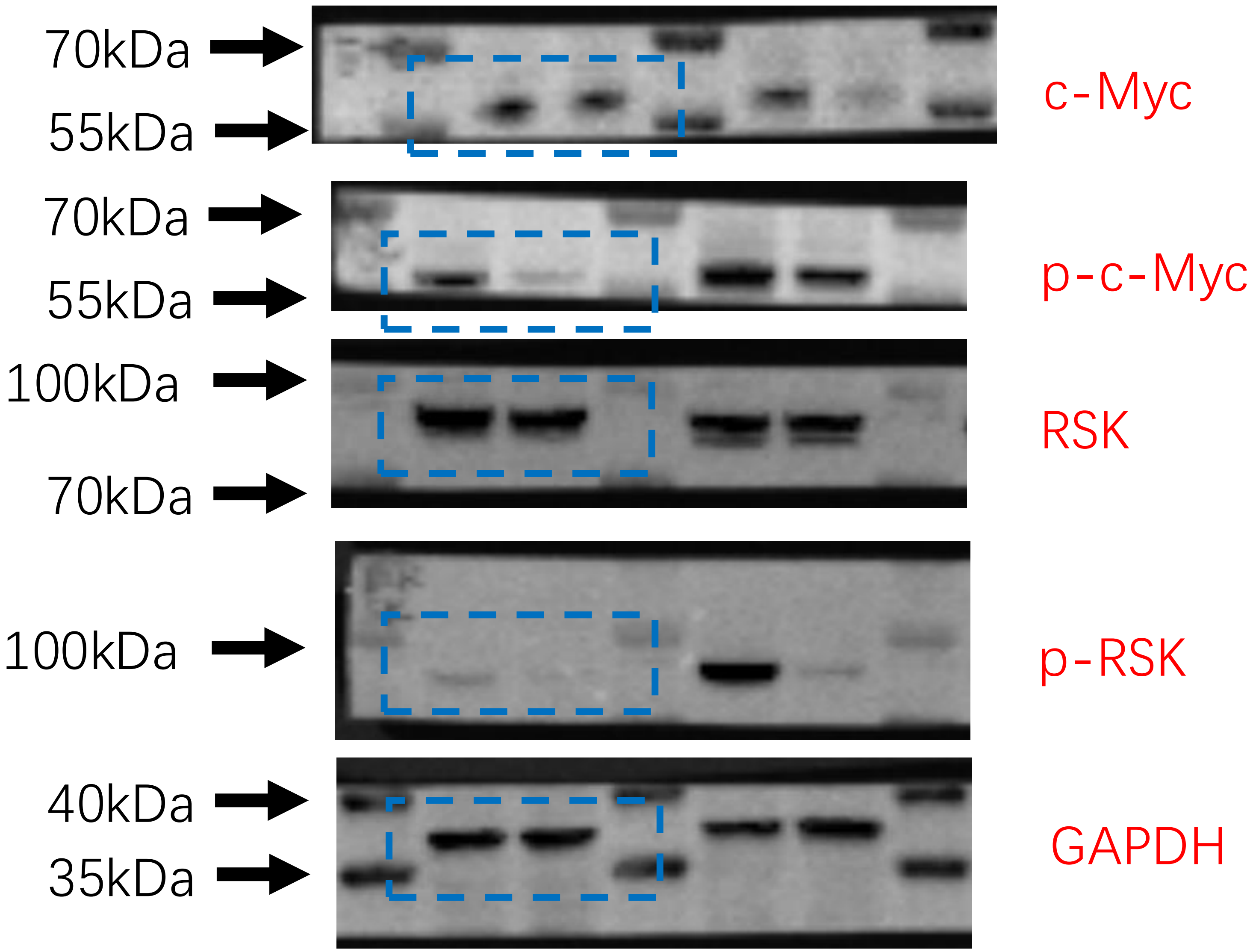

Full unedited gel for Supplementa
Figure 14F

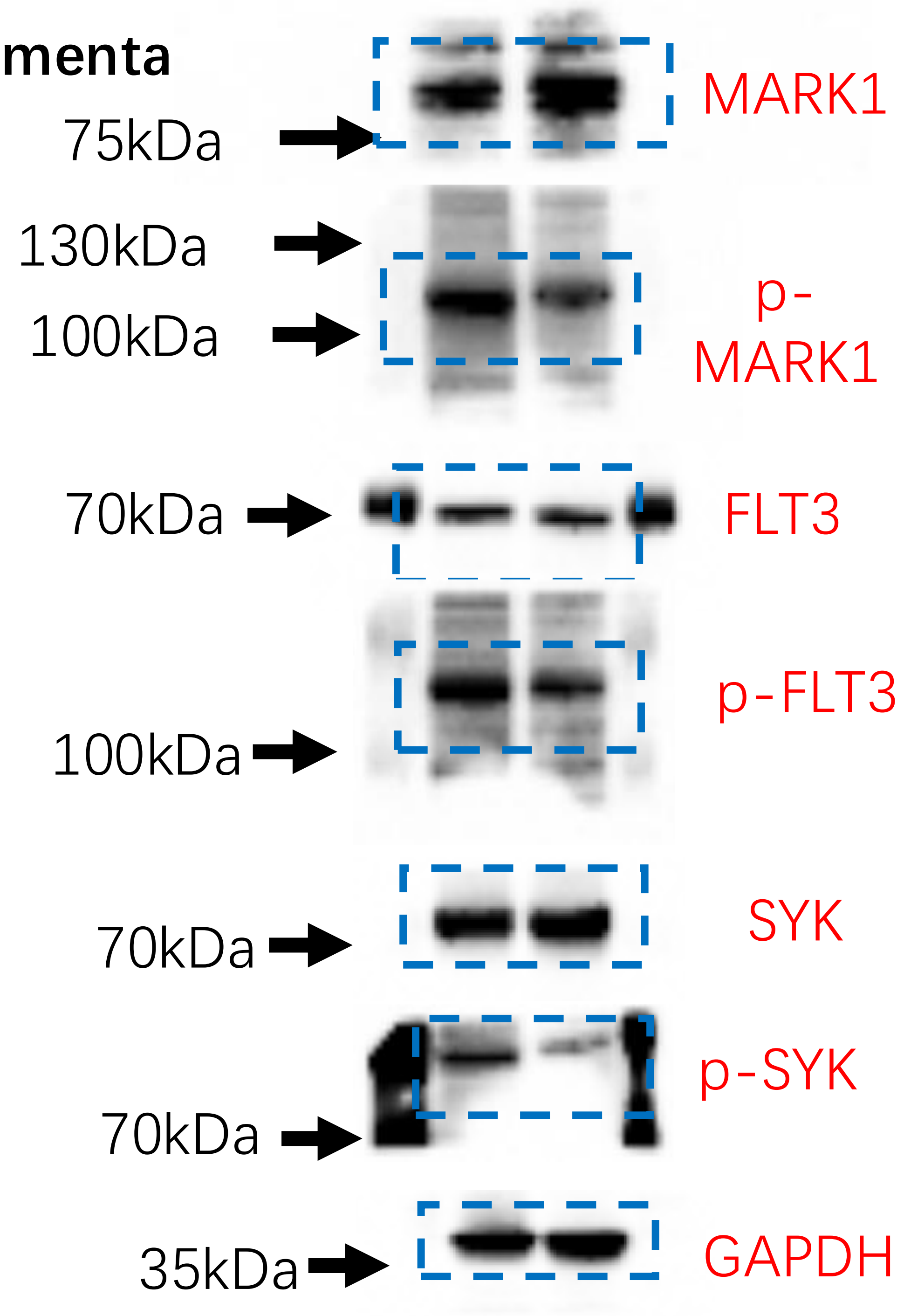

Supplement: Unedited blot and gel images [file jci-136-199497-s294.pdf]
